# *LINC00941* promotes pancreatic cancer malignancy by interacting with ANXA2 and suppressing NEDD4L-mediated degradation of ANXA2

**DOI:** 10.1038/s41419-022-05172-2

**Published:** 2022-08-18

**Authors:** Jie Wang, Zhiwei He, Xinyuan Liu, Jian Xu, Xueyi Jiang, Gang Quan, Jianxin Jiang

**Affiliations:** Department of Hepatobiliary Surgery, Renmin Hospital of Wuhan University, Wuhan, Hubei China

**Keywords:** Metastasis, Cell signalling, Pancreatic cancer, Ubiquitylation

## Abstract

Recently, long non-coding RNAs (lncRNA) have been proven to regulate pancreatic cancer (PC) progression. We aimed to explore the pathogenesis of *LINC00941* in PC regarding protein binding. By using PCR analysis, we found that *LINC00941* was overexpressed in PC tissues and was higher in patients with liver metastasis than in patients without liver metastasis. In addition, high *LINC00941* expression was associated with a poor prognosis. Functional experiments and mice models were respectively used to evaluate PC cell proliferation and migration in vitro and in vivo. The results suggested that *LINC00941* overexpression promoted PC proliferation and metastasis. Subsequently, RNA pull-down, mass spectrometry (MS), and RNA-binding protein immunoprecipitation (RIP) were performed to identify *LINC00941*-interacting proteins. The results suggested that ANXA2 was the potential *LINC00941*-interacting protein. Nucleotides 500–1390 of *LINC00941* could bind to the Annexin 1 domain of ANXA2. *LINC00941*-mediated malignant phenotype of PC was reversed by *ANXA2* depletion. Co-immunoprecipitation (Co-IP) followed by MS was conducted to determine the potential interacting protein of LINC00941. The results illustrated that NEDD4L, an E3 ligase involved in ubiquitin-mediated protein degradation, bound to the Annexin 1 domain of ANXA2 and promoted its degradation. Mechanically, *LINC00941* functioned as a decoy to bind to ANXA2 and suppressed its degradation by enclosing the domain that binds to NEDD4L. Eventually, *LINC00941* upregulated ANXA2 and activated FAK/AKT signaling, increasing PC cell proliferation and metastasis. This study indicates that *LINC00941* promotes PC proliferation and metastasis by binding ANXA2 and potentiating its stability, leading to the activation of FAK/AKT signaling. Our data demonstrate that *LINC00941* may serve as a novel target for prognosis and therapy.

## Introduction

Pancreatic cancer (PC) is a highly malignant solid tumor with high mortality among digestive tumors [1]. Due to mild early symptoms, difficult diagnosis, low resection rate, and high recurrence rate, the 5-year overall survival rate of patients is less than 8% [2]. However, the past decades of unremitting efforts and research have not significantly improved the therapeutic effect on PC [3]. Despite the continuous improvement in surgical methods, the prognosis has slightly improved. Meanwhile, patients with PC are often diagnosed at the advanced stages, and most patients lose the opportunity for surgery [4]. Some tumors have been identified through the detection of critical molecular markers, such as alpha fetal protein (AFP) in liver cancer [5], CA19-9 in PC [6]. However, when CA19-9 is used alone in the diagnosis of pancreatic cancer, negative results may result in missed diagnosis [7]. Therefore, it is extremely urgent to identify more effective molecular markers for the diagnosis and treatment of PC.

Currently, long non-coding RNA (lncRNA) is a hot spot in tumor marker research and has been validated in many tumors as a molecular target [8]. There are many studies on PC-associated lncRNAs, and the current focus is mainly on their tumor-promoting or tumor-suppressing biological effects [9]. Some studies indicate that lncRNAs may be used as an evaluation index for the malignant progression, early detection or prognostic evaluation of PC [10]. In previous studies, we identified a critical lncRNA, *LINC00941*, which is upregulated in PC and promotes the proliferation, invasion, and metastasis of PC [11]. Multiple studies have reported *LINC00941* as an oncogene in PC, participating in the regulation of cell proliferation, metastasis, and metabolism [12, 13]. However, the molecular mechanism of *LINC00941* in the malignant progression of PC has not been clearly elucidated.

LncRNAs exert their biological functions through a number of mechanisms, among which the competing endogenous RNA (ceRNA) mechanism and its scaffolding function are the most reported. *LINC00941* is known as a sponge for miRNAs, including *miR-877-3p* and *miR-335-5p* in esophageal squamous cell carcinoma and PC [11, 14]. However, whether *LINC00941* acts as a scaffold or decoy to regulate protein function remains unclear. LncRNA diffuses throughout the nucleus and cytoplasm, and it can interact with nearby proteins to mediate the expression, function, activity, and location of its target protein [15]. LncRNAs may regulate the biological process of cancer through its influence on post-translational protein modification, and protein ubiquitination is a critical post-translational modification involved in lncRNA-medicated multiple cellular processes [16–18]. Lu et al. find that *Snhg6* can regulate the ubiquitination of EZH2 to mediate MDSC differentiation [19]. Moreover, *SNHG17* can recruit LRPPRC to obstruct c-Myc ubiquitination and thus promote c-Myc expression and proliferation [20]. Therefore, lncRNA-mediated downstream protein ubiquitination is one of the possible molecular mechanisms for tumorigenesis and development.

ANXA2 is a member of the calcium-mediated phospholipid-binding protein family, which mediates many aspects of intercellular and extracellular microenvironment communications and cell survival [21]. Recently, accumulated studies suggest that ANXA2 is involved in the metastasis of several types of cancer, including gastric cancer [22], colorectal cancer [23], prostate cancer [24], and breast cancer [25]. In addition, ANXA2 may act as a potential marker of immunosuppression to regulate the infiltration of tumor-related macrophages, regulatory T cells, and myeloid-derived suppressor cells [26]. Moreover, a previous report indicates that lncRNA MIR155HG participates in the regulation of ANXA2 and induces M2 macrophage polarization and drug resistance of colorectal cancer [27].

To identify the function and mechanism of *LINC00941*, we analyzed the expression and clinical correlation of *LINC00941* in PC and found that it was upregulated and positively correlated with poor prognosis. Furthermore, we screened its interacting protein ANXA2 and explored the role of its ubiquitination in PC progression. We found that *LINC00941* most likely competitively bound to ANXA2 and blocked the interaction region of an E3 ubiquitin ligase, NEDD4L, to inhibit ANXA2 degradation, thus activating the downstream FAK and AKT signaling pathways to enhance PC malignancy.

## Materials and methods

### Human tissues samples

Six samples of PC tissues were obtained by surgical resection with the consent of patients. All the samples were collected at Renmin Hospital of Wuhan University and stored at −80 °C. The experiments using these samples were approved by the ethics committee of Renmin Hospital of Wuhan University.

### Cell lines and cell culture

All cell lines were purchased from the American Type Culture Collection. Among them, human pancreatic ductal epithelial (HPDE) cells and PC cell lines including AsPC-1, BxPC-3, and Capan-2 were cultured in RPMI-1640 supplemented with 10% fetal bovine serum (FBS). 293T cells and PC cells, including MIA PaCa-2 and PANC-1, were cultured in DMEM supplemented with 10% FBS. All cell lines were incubated at 37 °C in 5% CO_2_.

### Plasmids, siRNA, and stable cell line construction

Myc-ubiquitin (wild type, K48) plasmid was purchased from Genechem (Shanghai, China). FLAG-ANXA2 and HA-NEDD4L plasmids were purchased from Hanbio (Shanghai, China). *NEDD4L* siRNA was purchased from Ribobio (Guangzhou, China). Lentiviral vectors for *LINC00941* overexpression (lv-LINC00941) and knockdown (sh-LINC00941) and *ANXA2* knockdown (sh-ANXA2) were purchased from Genechem. Plasmids and siRNAs were transiently transfected into PC cells using Lipofectamine 3000 (Invitrogen, USA). Lentiviral vectors were stably transfected into PC cells with HitransG P (Genechem). All transfection methods were performed according to the manufacturer's protocols.

### Real-time fluorescence quantitative PCR (RT-qPCR) analysis

Total RNA of PC cell lines and tissues was extracted using RNA-easy Isolation Reagent (R701-01/02; Vazyme, China). The RNA was reverse-transcribed into cDNA using a HiScript III 1st Strand cDNA Synthesis Kit (+gDNA wiper) (R312-01; Vazyme). The cDNA was added to pre-mixed solution with ChamQ Universal SYBR qPCR Master Mix (Q711-02; Vazyme) followed by mixing with primers specific to *LINC00941*, *ANXA2*, and *GAPDH*, as examined by RT-qPCR. All primers are shown in Supplementary Table 1. The relative mRNA levels of the above genes were normalized against *GAPDH* and calculated using the 2 (^−△△^CT) method.

### Cell proliferation assays

Cell proliferative ability was measured by CCK8 assay using a Cell Counting Kit 8 (CCK-8) Kit (Dojindo Laboratories). PC cells were seeded into 96-well plates at a density of 3000 cells/well, and six parallel wells were set for every sample. Then the samples were incubated with CCK-8 reagent at 37 °C for 1 h. The cell proliferation rate was determined at 450 nm absorbance. For colony-formation assay, cells were seeded into six-well plates at a density of 500 cells/well and cultured for 2 weeks. Then the samples were fixed with 4% paraformaldehyde for 15 min at room temperature (RT). The fixed cells were stained using 3% crystal violet solution in six parallel wells for each sample and repeated in triplicate. Colonies were visualized by microscopy and counted by Image-J software.

### Cell migration and invasion assays

For wound-healing assays, PC cells were seeded into six-well plates and cultured until 80%–90% confluency. Then the cells in the middle of the wells were scratched with a 200-μL pipette tip. The medium was thereafter replaced with serum-free medium, and the cells were cultured for 24 h. The wound area was measured in five distinct fields of wound sites using a microscope and Image-J software. The wound gap percentage was calculated as the ratio of the residual wound area to the original wound area. For transwell migration assays, 5 × 10^4^ PC cells were seeded into the upper chambers (BD BioCoat, USA) with 200 μL serum-free medium, while the lower chamber contained medium with 10% FBS. After 36 h of cell culture, the migrated cells in the bottom of the upper chamber were fixed with 4% paraformaldehyde, followed by staining with 0.1% crystal violet, and counted by microscopy. For transwell invasion assays, the upper chamber was coated with Matrigel (BD Biosciences, USA), and 5 × 10^4^ PC cells were plated in the upper chamber and cultured in 200 μL serum-free medium while the lower chamber contained 700 μL medium with 10% FBS. The subsequent steps were the same as the migration assay. Five randomly selected fields of cells were counted for both the migration and invasion assays, and assays were performed in triplicate.

### Western blotting

Total protein was extracted from cells using RIPA lysis buffer containing protease inhibitors, phosphatase inhibitors, and PMSF (Boster, China). The samples were quantified by BCA assay (Boster, China) and boiled in SDS loading buffer. Then the extracted protein was loaded onto SDS-PAGE gels, and electrophoresis was performed at 80 V for 2 h in the Bio-Rad Electrophoresis System (Bio-Rad). Next, the membranes were blocked for 2 h at RT with blocking buffer. Membranes were incubated with corresponding primary antibodies overnight at 4 °C, followed by secondary antibodies (1:2000; SA00001-1/SA00001-2, Proteintech). Protein–antibody complexes were visualized using an ECL Enhanced Kit (RM00021; ABclonal, China). Primary antibodies against GAPDH (1:1000; 10494-1-AP, Proteintech), β-actin (1:1000; 20536-1-AP, Proteintech), ANXA2 (1:1000; 11256-1-AP, Proteintech), NEDD4L (1:1000; 13690-1-AP, Proteintech), Myc-tag (1:1000; 16286-1-AP, Proteintech), HA-tag (1:1000; 51064-2-AP, Proteintech), FLAG-tag (1:1000; 66008-3-Ig, Proteintech), FAK (1:1000; 12636-1-AP, Proteintech), p-FAK Y397 (1:1000; ab81298, Abcam), AKT (1:1000; 10176-2-AP, Proteintech), and p-AKT Ser473 (1:1000; 66444-1-Ig, Proteintech) were used.

### RNA pull-down assay

The linearized target DNA was obtained using *ApaI* restriction enzyme plasmid, and the transcription of *LINC00941* was synthesized by a Transcript Aid T7 High Yield Transcription Kit (K0441; Thermo). The RNA pull-down assay was performed using a Pierce Magnetic RNA-protein pull-down Kit (20164; Thermo) according to the manufacturer’s protocol. Briefly, the obtained RNA was biotinylated, and biotin-labeled RNA was incubated with streptavidin magnetic beads and RNA capture buffer for 30 min at RT with rotation. Next, the sediment was separated using a magnetic separator and mixed with 100 µL Protein–RNA Binding Buffer, and the mixture was incubated for 60 min at RT. Subsequently, the sample protein was extracted and mixed with the biotinylated RNA mixture and SDS loading buffer, and the final mixture was heated for 5 min at 95 °C, whereupon it was then detected by western blot assay.

### RNA-binding protein immunoprecipitation (RIP) assay

Complete RIP lysate buffer was prepared using a Magna RIP RNA-Binding Protein Immunoprecipitation Kit (17-700; Millipore, USA). The cells in the exponential stage of proliferation were selected, digested, and collected by trypsinization. The collected cells were centrifuged for 5 min at 4 °C and washed with pre-cooled PBS. After centrifugation, the precipitate was added to the complete RIP lysate buffer and incubated on ice for 5 min. Then, 50 µL magnetic beads were conjugated with anti-ANXA2 antibodies (Proteintech) or anti-IgG (Abcam) and incubated at RT for 30 min. Subsequently, the cell suspension was quickly dissolved and centrifuged at 15,000 rpm for 10 min at 4 °C. Then, 100 µL supernatant was added to the antibody beads, and the mixture was incubated overnight at 4 °C with rotation. The cell suspension was quickly dissolved and centrifuged at 15000 rpm for 10 min at 4 °C. Next, the samples were separated by proteinase K and DNase I. Finally, the supernatant was purified by chloroform isopropyl alcohol and anhydrous ethanol, and the extracted RNA was detected by qRT-PCR.

### Co-immunoprecipitation (co-IP)

Whole-cell protein lysates were obtained using IP lysis buffer (1.0% NP-40 lysis buffer, 0.2 mM EDTA, 20 mM pH 8.0 Tris-HCl, 180 mM NaCl), phosphatase inhibitors (Thermo), and protease inhibitor cocktail (Roche) at 4 °C. After centrifugation at 12000 rpm for 10 min at 4 °C, the supernatants were prepared for endogenous IP or exogenous IP. For endogenous IP, the supernatants were incubated with corresponding antibodies and Protein A/G Magnetic Beads (MCE) at 4 °C overnight. For exogeneous IP, supernatants were incubated with anti-FLAG or anti-HA antibodies and Protein A/G Magnetic Beads (MCE) at 4 °C overnight. Subsequently, the samples were separated by a magnetic separator and denatured by SDS loading buffer, and examined by western blotting or mass spectrum analysis.

### Immunofluorescence (IF) staining

PC cells were seeded in a sterilized glass slide and fixed with 4% paraformaldehyde for 10 min at RT. Then the samples were permeabilized with 0.1% Triton X-100 for 5 min and blocked with 2% fetal bovine albumin for 30 min at RT. The cells were incubated with ANXA2 antibody at 4 °C overnight. After washing with PBS buffer, the samples were incubated with fluorescent secondary antibody and *LINC00941* FISH probe (Ribobio, China) for 1 h at RT. Next, cells were rinsed with PBS buffer and incubated with DAPI staining solution for 10 min at RT. Images were visualized by confocal laser scanning microscopy (Zeiss, LSM700).

### Animal models

Six-week-old BALB/c nude mice were randomly separated into different groups with correlative treatment. Stable cell lines (PANC-1) expressing lv-LINC00941, vector, or lv-LINC00941 + sh-ANXA2 were generated, and a lentiviral vector containing luciferase probe was co-transfected into stable cell lines for in vitro imaging of the metastasis model. Stable PANC-1 cells (5 × 10^6^) were subcutaneously injected into the armpits of mice for the proliferation model and injected into the spleen through an opening in the abdomen under anesthesia for the metastasis model. After tumor formation, the mice underwent treatment with PF-562271 (30 mg/kg/d) in an oral form. For the proliferation model, tumor volumes were monitored every 3 days for 1 month. The mice were then killed, and tumors were excised and weighed. Tumor volumes were calculated using the following formula: V = (L × W^2^)/2, where V = volume (mm^3^), L = length (mm), and W = width (mm). For the metastasis model, the mice underwent in vitro imaging every week. The livers and lungs of mice were dissected and measured by section observation under microscopy. All tumors, livers, and lungs were paraffin-embedded and sliced into sections followed by immunohistochemistry and H&E staining, and were then observed by microscopy. The animal experiments were approved by the Animal Research Ethics Committees of Renmin Hospital of Wuhan University.

### Bioinformatic analysis

Gene expression data of GSE101094 were obtained from Gene Expression Omnibus (GEO) website and differential gene expression analysis was performed using the R package limma. The significant DEGs (FDR < 0.05) were visualized by heat maps and volcano plot. Using the RNA sequencing data from TCGA-PAAD dataset, we ranked the gene list by the value of log_2_FC, and enrichment analysis (GSEA) was performed on “LINC00941 high” versus “LINC00941 low” samples to define differentially expressed pathways.

### Statistical analysis

Statistical analysis was performed using GraphPad Prism 8.0 software (La Jolla, USA) and SPSS 22.0 software (IBM Corp, USA). Student’s *t* test or analysis of variance was used to compare the mean differences of continuous variables. Kaplan–Meier estimates were calculated and compared using log-rank tests. The Cox proportional risk regression model was used to assess the prognostic variables of overall survival (OS) and disease-free survival (DFS). *P* values less than 0.05 were considered significant.

## Results

### *LINC00941* expression is elevated in PC and is correlated with malignant progression and poor prognosis

**Fig. 1:**
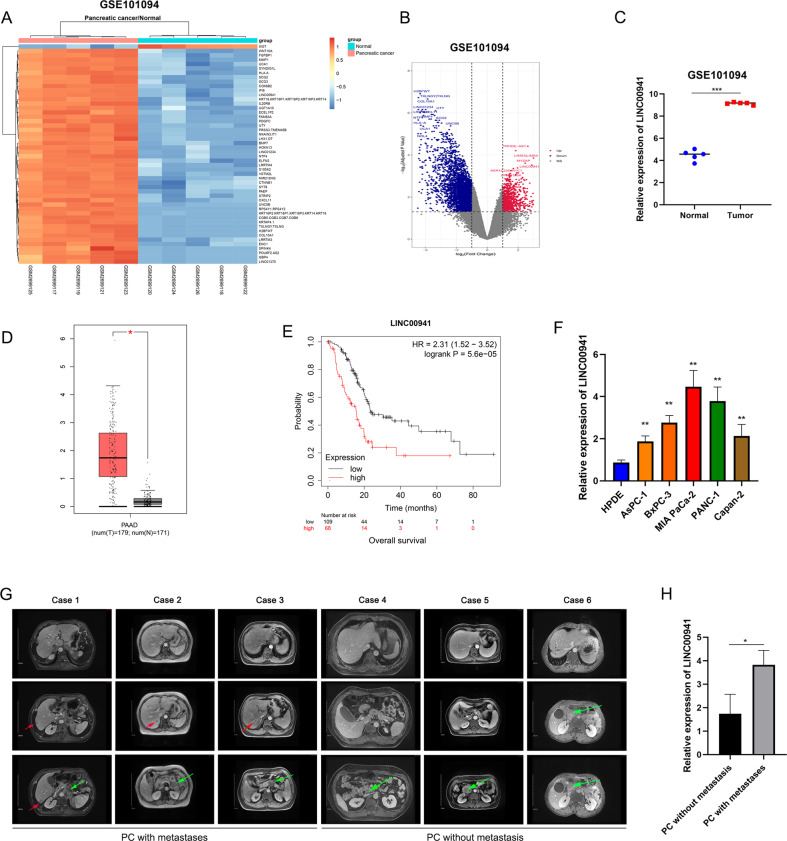
Screening and identification of lncRNA in pancreatic cancer. **A** Heatmap of different RNA at the transcriptional level from five paired pancreatic cancer tissues and adjacent normal tissues based on GEO database (GSE101094). **B** Volcano plot of differentially expressed genes in GSE101094. **C** The expression of lncRNA LINC00941 in pancreatic cancer tissues compared to adjacent normal tissues in GSE101094. **D** The expression of LINC00941 in pancreatic cancer tissues and normal tissues in GEPIA based on TCGA database (tumors tissues:179, normal tissues: 171). **E** Kaplan–Meier curve was performed to analyze the prognosis of pancreatic cancer patients with high LINC00941 expression compared to low LINC00941 expression. **F** The expression of LINC00941 in HPDE and five pancreatic cancer cell lines, including AsPC-1, BxPC-3, MIA PaCa-2, PANC-1, and Capan-2 were validated by qRT-PCR analysis. **G** The representative MRI images of pancreatic cancer patients with or without isolated liver metastases. Case 1-3 had isolated liver metastases, and Case 4-6 had no liver metastasis. Red arrow points to liver metastases, green arrow points to the primary tumor of the pancreas. **H** The expression of LINC00941 in pancreatic cancer tissues from patients with metastasis and without metastasis was evaluated by RT-qPCR analysis. *, **, and ***, respectively, represent *P* < 0.05, *P* < 0.01, and *P* < 0.001.

First, we analyzed lncRNA expression profiles in human PC tissues and adjacent non-cancerous tissues using the GEO database (GSE101094). A list of the top 50 genes with the most significant differences is shown in Fig. 1A. A volcano map in Fig. 1B shows the distribution of differential genes. Among them, *LINC00941* was one of the most differential lncRNAs and its expression was high in PC tissues (Fig. 1C). To clarify the expression of *LINC00941* in PC, we then used GEPIA database (http://gepia.cancer-pku.cn/), which is based on TCGA database to obtain the relative expression between PC tissues and adjacent normal tissues. The result suggested that *LINC00941* was highly expressed in PC tissues (Fig. 1D). Subsequently, we discussed the effect of *LINC00941* expression on the PC patient survival rate based on TCGA database. We found that high *LINC00941* expression was positively associated with poor prognosis (Fig. 1E). Meanwhile, we designed subgroup analysis to describe overall survival according to different tumor stage and grade, respectively. High *LINC00941* expression in stage I–II and grade 1–2 also supported that high *LINC00941* expression was associated with unfavorable prognosis (Supplementary Fig. 1A–D). However, there was no difference between patients with high LINC00941 expression or low LINC00941 in grade 3 level of pancreatic cancer (Supplementary Fig. 1E). Due to the little number of patients in stage III-IV and grade 4, we did not analyze the results. Collectively, we performed univariate and multivariate Cox regression analyses using TCGA data and found that the expression of *LINC00941* was an independent prognostic factor in patients with PC (Supplementary Fig. 1F, G). To further confirm the expression of *LINC00941* in PC cells and tissues, we measured *LINC00941* expression in five PC cell lines, which revealed that *LINC00941* in PC cells was higher than in HPDE cells (Fig. 1F). We collected six clinical samples by surgical resection. Among them, three patients had distant metastasis that was mainly focused on the liver and surrounding lymph node metastasis, while the other three patients showed no signs of metastasis. Typical abdominal magnetic resonance imaging (MRI) images from these patients are presented in Fig. 1G. Subsequently, we measured the relative expression of *LINC00941* in PC with metastasis and PC without metastasis by PCR analysis. The results indicated that *LINC00941* expression was higher in PC with metastasis compared with PC without metastasis (Fig. 1H). Eventually, we explored the correlation of *LINC00941* and immune cell infiltration based on TCGA database, revealing that LINC00941 was positively correlated with macrophages M0 and M1; However, LINC00941 was negatively associated with B cells naive, plasma cells, T cells CD8, and T cells follicular helper (Supplementary Fig. 1H).

### *LINC00941* can directly interact with ANXA2

**Fig. 2:**
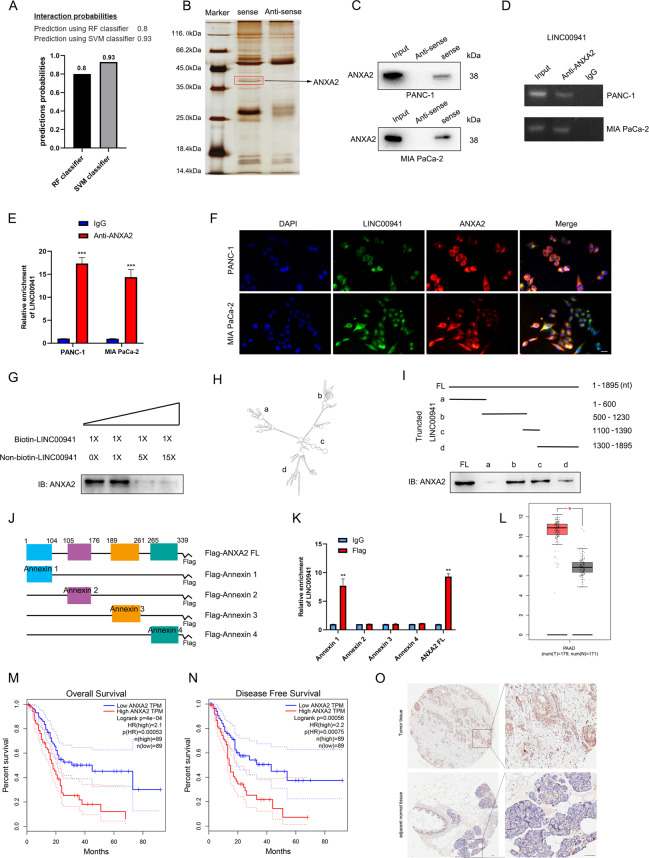
LINC00941 can interact with ANXA2 protein. **A** Bioinformatic prediction (RNA–Protein Interaction Prediction, RPISeq) was performed to predict the probability of the interaction between LINC00941 and ANXA2 by analyzing their sequences. Interaction probabilities generated by RPISeq range from 0 to 1. In performance evaluation experiments, predictions with probabilities >0.5 were considered “positive”. **B** Silver-stained SDS-PAGE gel of proteins immunoprecipitated by the sense and antisense of LINC00941. The differentially exhibited lanes were used for the mass spectrum. Among them, ANXA2 was one of the potential binding protein. **C** Immunoblotting for specific correlation of ANXA2 with LINC00941 from RNA pull-down assays. **D** RNA-binding protein immunoprecipitation (RIP) assay was further used to validate the interaction between ANXA2 and LINC00941, followed by agarose gel electrophoresis. **E** RIP assay for ANXA2 followed by qRT-PCR analysis. **F** Immunofluorescence was performed to clarify the co-location of LINC00941 and ANXA2. **G** Competitive RNA pull-down assays for LINC00941 followed by immunoblotting analysis. **H** The secondary structure of LINC00941 was simulated via importing the sequence of LINC00941 into ViennaRNA. **I** According to the secondary structure of LINC00941, the relevant full-length and truncated LINC00941 were constructed. RNA-pull-down assay for full-length and truncated LIN00941 followed by immunoblotting analysis. **J** The schematic diagram of full-length and truncated ANXA2. **K** RIP assay for full-length and truncated ANXA2 followed by qRT-PCR analysis. **L** The expression of ANXA2 in pancreatic cancer tissues compared to normal tissues based on the TCGA database. **M**, **N** Kaplan–Meier curve respectively evaluated the overall survival and disease-free survival of 178 pancreatic cancer patients based on the TCGA database. **O** The expression of ANXA2 in pancreatic cancer tissues and adjacent normal tissues by IHC analysis. *, **, and ***, respectively, represent *P* < 0.05, *P* < 0.01, and *P* < 0.001.

One of the main functions of lncRNAs is that they can bind to proteins and exert multiple biological functions, thereby acting as signals, decoys, guides, and scaffolds [28]. To investigate the potential binding protein of *LINC00941*, we first searched the bioinformatics prediction site RNAInter (http://www.rnainter.org/) and found that ANXA2 was an *LINC00941*-interacting protein (Supplementary Fig. 2A). In addition, RNA–Protein Interaction Prediction (RPISeq) (http://pridb.gdcb.iastate.edu/RPISeq/) was performed to estimate the interaction probabilities between *LINC00941* and ANXA2, the results of which confirmed their interaction (Fig. 2A). To identify whether *LINC00941* could bind proteins, we used RNA pull-down assays followed by mass spectrometry (MS) analysis to identify potential interacting proteins (Fig. 2B). The results suggested that ANXA2 was an interacting protein (Supplementary Fig. 2B). In addition, we performed RNA pull-down assays followed by western blotting to clarify the association between ANXA2 and *LINC00941*. The results were consistent in that ANXA2 was pulled down in the biotin-labeled sense *LINC00941* group (Fig. 2C). To further confirm this interaction, RIP assay followed by agarose gel electrophoresis and qRT-PCR analysis were performed, and the results also supported that *LINC00941* was enriched by ANXA2 (Fig. 2D, E). Furthermore, *LINC00941* and ANXA2 were mainly co-located in the cytoplasm (Fig. 2F). Meanwhile, competitive RNA pull-down assays also suggested *LINC00941* could bind ANXA2 (Fig. 2G). Subsequently, we imported the sequence of *LINC00941* into ViennaRNA (http://rna.tbi.univie.ac.at/) to observe its secondary structure (Fig. 2H). To reveal the exact regions of *LINC00941* that ANXA2 might bind, we constructed a series of truncated variants of *LINC00941* with reference to the secondary structure and used RNA pull-downs followed by western blotting to clarify the interaction regions. The result illustrated that nucleotides 500–1390 of *LINC00941* could bind ANXA2 (Fig. 2I). Conversely, we also constructed full-length ANXA2 and different truncated variants with reference to different domains according to Uniprot (https://www.uniprot.org/) (Fig. 2J). RIP assay demonstrated that *LINC00941* could be enriched in the Annexin 1 domain of ANXA2 and full-length ANXA2 (Fig. 2K). In an analysis of the expression of *ANXA2* in PC and normal tissues by Gene Expression Profiling Interactive Analysis (GEPIA, http://gepia.cancer-pku.cn/), we found *ANXA2* expression was increased and low expression of *ANXA2* was associated with a better prognosis (Fig. 2L–N). In addition, IHC assay also suggested ANXA2 was overexpressed in PC tissues compared with normal tissues (Fig. 2O). Furthermore, PC cell lines showed higher ANXA2 expression than HPDE cells by western blotting and PCR analysis (Supplementary Fig. 2C, D).

### *LINC00941* upregulates the protein expression of ANXA2 by suppressing its ubiquitination

**Fig. 3:**
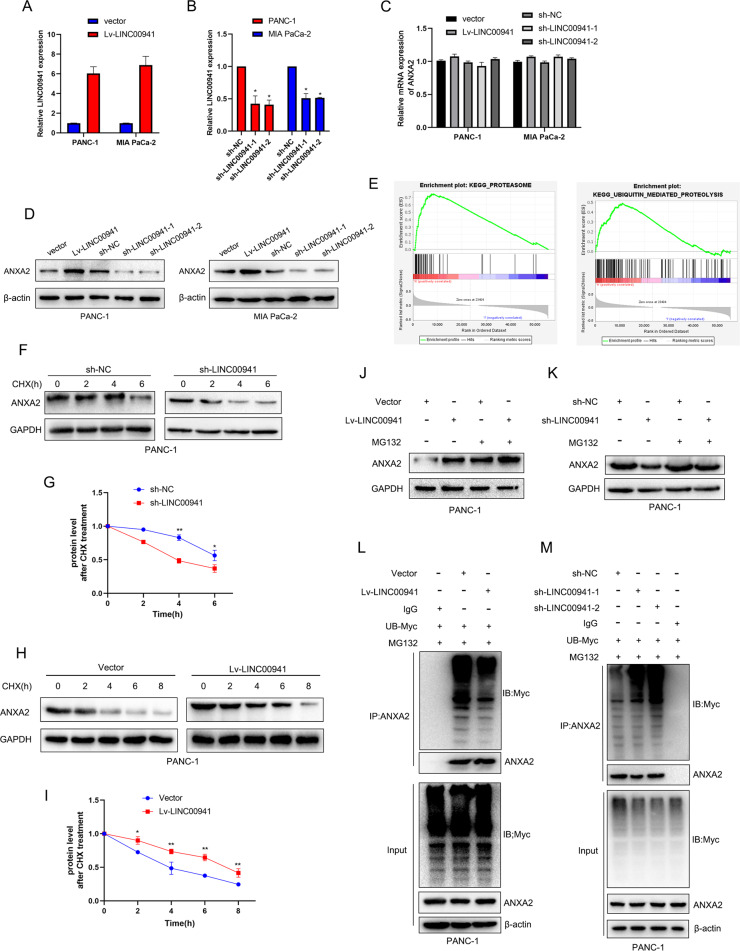
LINC00941 upregulates the protein expression of ANXA2 by suppressing its ubiquitination. **A**, **B** The transfection efficiency of LINC00941 overexpression and knockdown was validated by qRT-PCR analysis. **C** The mRNA expression level of ANXA2 was measured by qRT-PCR analysis. **D** The protein expression level of ANXA2 was measured by western blot analysis. **E** GSEA analysis based on the TCGA database was performed to predict that LINC00941 was positively associated with proteasome and ubiquitin-mediated proteolysis. **F**–**I** Protein stability assay by using cycloheximide (CHX, 50 μg/mL) to treat cells at the different time was performed to evaluate the effect of LINC00941 overexpression (**F**, **G**) or knockdown (**H**, **I**), followed by western blot analysis. **J**, **K** Western blot analysis of PANC-1 cells stably transfected with LINC00941 overexpression or knockdown and treated with the proteasome inhibitor MG132 (10 μM). **L** The ubiquitination of ANXA2 upon LINC00941 or not were analyzed by Co-IP with the treatment of MG132 and Myc-Ubi. **M** The ubiquitination of ANXA2 in LINC00941 knockdown or negative control group was analyzed by Co-IP with the treatment of MG132 and Myc-Ubi. * and **, respectively, represent *P* < 0.05 and *P* < 0.01.

Previous results confirmed that *LINC00941* was highly expressed in PC cells. Given that *LINC00941* expression in PANC-1 and MIA PaCa-2 was highest among the PC cell lines, we selected them as the experimental cell lines. We clarified that *LINC00941* could interact with ANXA2; however, it was not clear whether *LINC00941* could affect the expression of ANXA2. Therefore, we first constructed stably transfected cell lines with *LINC00941* overexpression (Lv-LINC00941) or knockdown lentiviral vector (sh-LINC00941). PCR analysis was performed to confirm that the transfection was successful (Fig. 3A, B). Then, we measured the mRNA level of ANXA2 when we overexpressed or knocked down *LINC00941* by PCR analysis. However, there was no significant difference between the overexpression and knockdown groups (Fig. 3C). Subsequently, western blotting analysis was performed to detect the ANXA2 changes. Notably, *LINC00941* overexpression and knockdown remarkably altered the expression of ANXA2 compared with that of the control groups (Fig. 3D). To elucidate the mechanism by which *LINC00941* positively upregulated the protein expression of ANXA2, we performed gene set enrichment analysis (GSEA) based on TCGA to identify the potential biological function of *LINC00941*. GSEA showed that *LINC00941* was positively associated with proteasome- and ubiquitin-mediated proteolysis (Fig. 3E). Ubiquitination is a common post-translational modification that degrades target proteins through proteasome systems [29]. To verify this hypothesis, the cell samples were treated with cycloheximide (CHX, 50 μg/mL) for different periods of time to examine the protein stability of ANXA2. Interestingly, *LINC00941* overexpression prolonged the half-life of ANXA2 degradation, while *LINC00941* knockdown decreased the stability of ANXA2 and accelerated its degradation (Fig. 3D–I). MG132, a protease inhibitor, can accurately inhibit the ubiquitin-mediated proteasome pathway. MG132 led to the accumulation of ANXA2 by inhibiting the proteosome pathway. When we upregulated *LINC00941* expression in the presence of MG132, ANXA2 expression was further upregulated by suppressing the ubiquitin-mediated proteasome pathway (Fig. 3J). Meanwhile, knockdown of *LINC00941* expression inducing ANXA2 downregulation was significantly reversed by MG132 treatment, implying that *LINC00941* was involved in ANXA2 degradation by ubiquitination (Fig. 3K). To further investigate the effect of *LINC00941* on the ubiquitination of ANXA2, we measured the ubiquitination levels when upregulating or downregulating the expression of *LINC00941*. The results suggested that *LINC00941* overexpression remarkably decreased the ubiquitination of ANXA2; however, ANXA2 ubiquitination was increased by *LINC00941* depletion (Fig. 3L, M). Collectively, these results revealed that *LINC00941* upregulates the protein expression of ANXA2 by suppressing its ubiquitination.

### *LINC00941* accelerates proliferation, migration, and invasion by elevating the expression of ANXA2 in PC cells

**Fig. 4:**
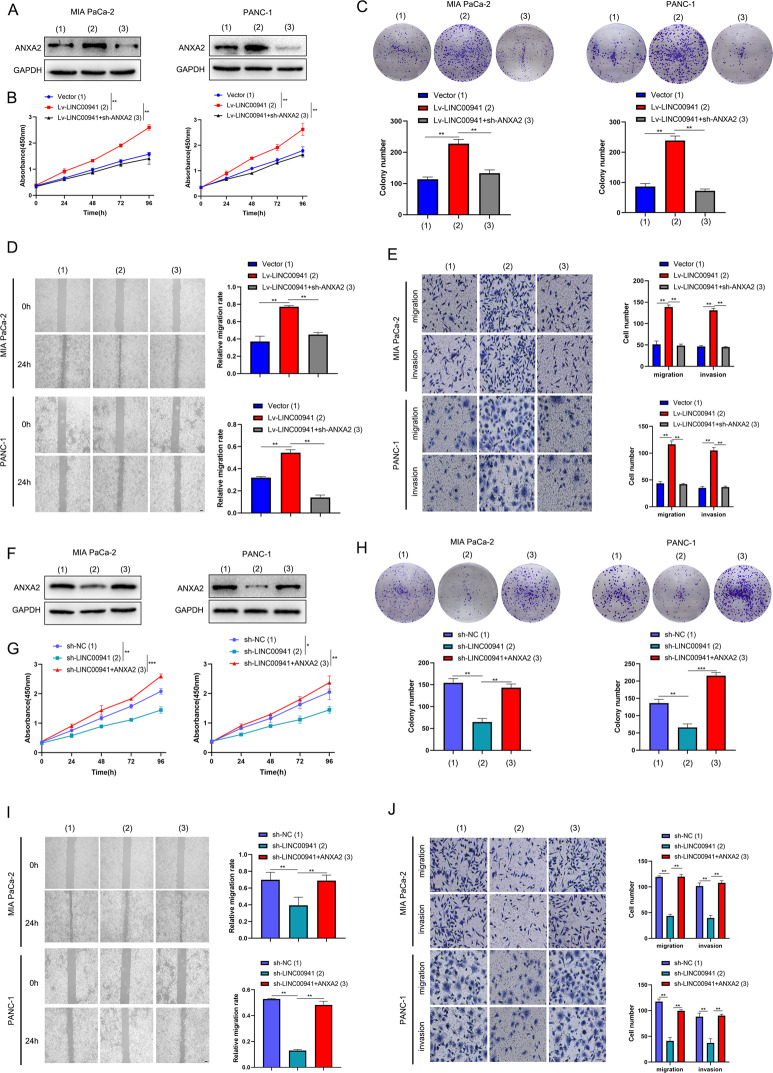
LINC00941 accelerates proliferation, migration, and invasion through elevating the expression of ANXA2 in PC cells. **A** The protein expression of ANXA2 in LINC00941 overexpression (lv-LINC00941) group, lv-LINC00941 + sh-ANXA2 group, and empty vector group was evaluated by western blot analysis. **B** The cell viability was measured in the empty vector group, lv-LINC00941 group, and lv-LINC00941 + sh-ANXA2 group by CCK-8 assay. **C** The colony-formation assay was performed to clarify the proliferation ability of pancreatic cancer cell in empty vector group, lv-LINC00941 group, and lv-LINC00941 + sh-ANXA2 group. **D** The cell migration ability was measured in the empty vector group, lv-LINC00941 group, and lv-LINC00941 + sh-ANXA2 group by wound-healing assay. **E** The cell migration and invasion were detected in the empty vector group, lv-LINC00941 group and lv-LINC00941 + sh-ANXA2 group by transwell assay. **F** The protein expression of ANXA2 in LINC00941 knockdown (sh-LINC00941) group, sh-LINC00941 + ANXA2 group and negative control group was evaluated by western blot analysis. **G** The cell viability was measured in the negative control group, sh-LINC00941 group, and sh-LINC00941 + ANXA2 group by CCK-8 assay. **H** The colony-formation assay was performed to clarify the proliferation ability of pancreatic cancer cell in the negative control group, sh-LINC00941 group, and sh-LINC00941 + ANXA2 group. **I** The cell migration ability was measured in the negative control group, sh-LINC00941 group, and sh-LINC00941 + ANXA2 group by wound-healing assay. **J** The cell migration and invasion were detected in negative control group, sh-LINC00941 group and sh-LINC00941 + ANXA2 group by transwell assay. *, **, and ***, respectively, represent *P* < 0.05, *P* < 0.01, and *P* < 0.001.

To explore the potential function of *LINC00941* and ANXA2 in PC progression, we first infected cells with *LINC00941* overexpression and knockdown lentivirus vectors and their control (Vector, shNC). Then the infected cells were subjected to cell proliferation, migration, and invasion analyses. Among them, *ANXA2* silencing or overexpression was performed to combine to evaluate the rescue effect of ANXA2 on *LINC00941*-mediated biological function. Western blot analysis indicated ANXA2 was upregulated in the Lv-LINC00941 group and could be reversed by *ANXA2* depletion (Fig. 4A). CCK-8 and colony-formation assay were performed to evaluate PC proliferation and revealed that *LINC00941* dramatically promoted PC cell division, while this effect could be blocked by *ANXA2* depletion (Fig. 4B, C). Wound-healing assay and transwell assay were used to evaluate migration and invasion, illustrating that these were enhanced in the Lv-LINC00941 groups but reversed in the Lv-LINC00941 + sh-ANXA2 group (Fig. 4D, E). Meanwhile, knockdown of *LINC00941* induced a reduction in ANXA2 protein but ANXA2 overexpression substantially reversed its expression in the sh-LINC00941 group (Fig. 4F). In contrast to *LINC00941* overexpression, cell proliferation was significantly inhibited in the sh-LINC00941 group in CCK-8 and colony-formation assays (Fig. 4G, H). In addition, cell migration and invasion were also suppressed in the sh-LINC00941 group by wound-healing and transwell assays. Interestingly, ANXA2 overexpression also dramatically rescued the inhibition by *LINC00941* depletion (Fig. 4I, J). Taken together, these data demonstrated that *LINC00941* accelerated proliferation, migration, and invasion by suppressing ANXA2 expression in PC cells.

### *LINC00941* acts as a decoy to competitively bind ANXA2 and inhibit NEDD4L-mediated ubiquitination

**Fig. 5:**
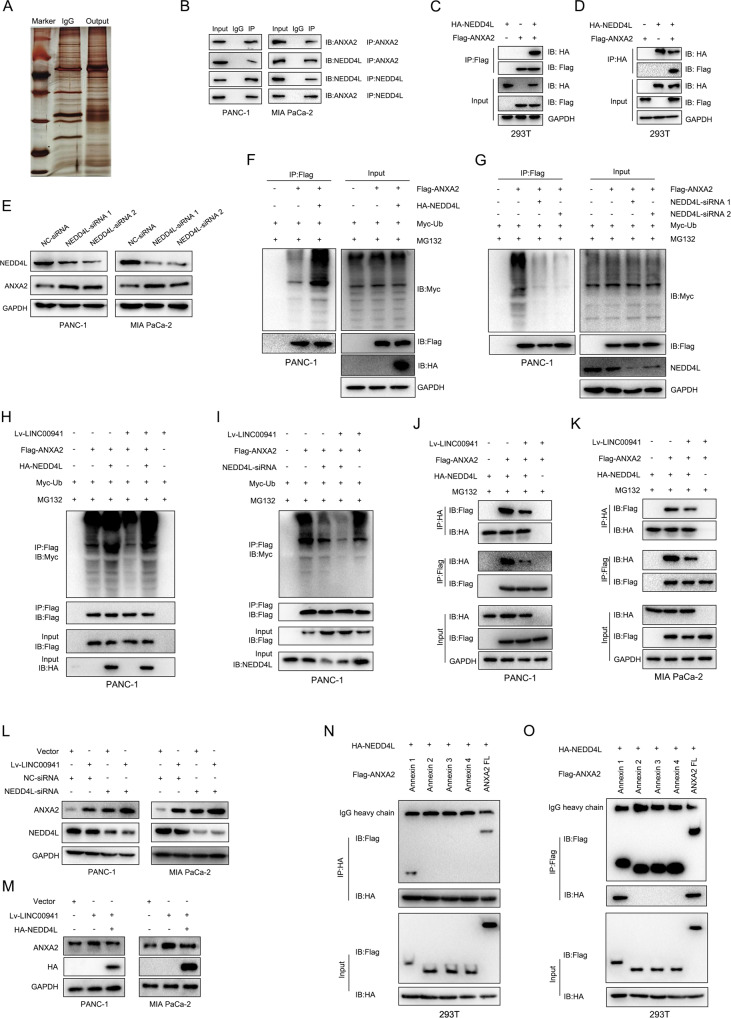
LINC00941 acts as a decoy to competitively bind ANXA2 and inhibits NEDD4L-mediated ubiquitination. **A** Silver-stained SDS-PAGE displayed a results of immunoprecipitated using IgG and anti-ANXA2 in ANXA2 overexpression PANC-1 cells, followed by mass spectrum. **B** Co-IP assay was performed to clarify the interaction between endogenous ANXA2 and endogenous NEDD4L in PANC-1 and MIA PaCa-2 cells. **C**, **D** Flag-tagged ANXA2 and HA-NEDD4L were transfected into 293T cells, followed by using Co-IP assay to clarify the interaction between exogenous NEDD4L and exogenous ANXA2. **E** The protein expression of NEDD4L and ANXA2 was measured in NEDD4L silenced group and negative control group by western blot. **F** The ubiquitination of ANXA2 upon NEDD4L or not were analyzed by Co-IP with the treatment of MG132 (10 μM) and Myc-Ubi. **G** The ubiquitination of ANXA2 in NEDD4L knockdown or not were analyzed by Co-IP with treatment of MG132 (10 μM) and Myc-Ubi. **H** The ubiquitination of ANXA2 in LINC00941 overexpression combining with NEDD4L or not were analyzed by Co-IP with the treatment of MG132 (10 μM) and Myc-Ubi. **I** The ubiquitination of ANXA2 in NEDD4L knockdown combining LINC00941 overexpression or not were analyzed by Co-IP with the treatment of MG132 (10 μM) and Myc-Ubi. **J**, **K** Co-IP assays were performed to clarify the effect of LINC00941 combining NEDD4L or not on the interaction between NEDD4L and ANXA2 with the treatment of MG132 (10 μM) in PANC-1 (**J**) and MIA PaCa-2 **K** cells. **L** The expression of ANXA2 was measured by western blot in LINC00941 overexpression combining NEDD4L knockdown in PANC-1 and MIA PaCa-2 cells. **M** The expression of ANXA2 was measured by western blot in LINC00941 overexpression combining NEDD4L overexpression in PANC-1 and MIA PaCa-2 cells. **N**, **O** The truncated of ANXA2 and NEDD4L was co-transfected into 293T cells, followed by Co-IP assay to clarify the interaction domain between ANXA2 and NEDD4L.

To further elucidate the in-depth mechanism of *LINC00941*-mediated PC progression, we focused our work on its interacting protein, ANXA2. We performed immunoprecipitation using anti-ANXA2 and IgG antibody by overexpressing ANXA2 in PANC-1 cells, followed by mass spectrometry analysis. The results showed that NEDD4L, an E3 ubiquitin ligase involved in the ubiquitin-mediated proteasome pathway, might be the potential interacting protein of ANXA2 (Fig. 5A and Supplementary Fig. 3A). Meanwhile, we used Ubibrowser (http://ubibrowser.ncpsb.org.cn/ubibrowser/) to predicate the potential interacting E3 ligases, including NEDD4L, which had a higher score, also supporting this presumption (Supplementary Fig. 3B). To further clarify this interaction between ANXA2 and NEDD4L, co-IP analysis was performed. The results suggested that endogenous ANXA2 interacted with NEDD4L in PC cells (Fig. 5B). Furthermore, we transfected HA-tagged NEDD4L and FLAG-tagged ANXA2 into 293T cells and revealed that exogenous NEDD4L also could interact with exogenous ANXA2 by co-IP analysis (Fig. 5C, D). Subsequently, we knocked down the expression of *NEDD4L* and evaluated whether it affected the expression of ANXA2. Interestingly, silenced *NEDD4L* enhanced ANXA2 expression, and NEDD4L was negatively associated with ANXA2 (Fig. 5E). To further elucidate whether NEDD4L-mediated ANXA2 downregulation participated in the ubiquitin-mediated proteasome pathway, we evaluated the levels of ANXA2 ubiquitination by overexpressing or silencing NEDD4L. The results indicated that NEDD4L overexpression dramatically promoted ANXA2 ubiquitination while *NEDD4L* knockdown significantly inhibited its ubiquitination (Fig. 5F, G).

Previous results suggested *LINC00941* could inhibit ANXA2 ubiquitination, while NEDD4L showed a stimulatory function. To reveal the underlying association between them, we transfected HA-tagged NEDD4L or *NEDD4L* siRNA into PANC-1 cells infected with Lv-LINC00941, which indicated that *LINC00941* could decrease ANXA2 ubiquitination degradation and partly protect ANXA2 from ubiquitination degradation by NEDD4L (Fig. 5H, I). Accumulating evidence proved that lncRNA acted as a decoy to adsorb miRNA and affect their regulation of expressed genes; or to sequester protein to regulate their function. Subsequently, we performed quantitative co-IP to verify that *LINC00941* is involved in the regulation of post-translational ubiquitin modification of ANXA2 by NEDD4L. The results indicated that *LINC00941* overexpression led to reduced interactions between NEDD4L and ANXA2 in PC cells with MG132 treatment (Fig. 5J, K). Meanwhile, we evaluated the effect of combining *LINC00941* and NEDD4L on ANXA2 expression. Notably, the expression of ANXA2 was increased by co-transfecting Lv-LINC00941 and *NEDD4L* siRNA in PANC-1 and MIA PaCa-2 cells (Fig. 5L). Nevertheless, NEDD4L overexpression could partly reverse the increased ANXA2 expression by *LINC00941* overexpression (Fig. 5M). To further identify the in-depth molecular mechanism by which *LINC00941* affected the interactions between NEDD4L and ANXA2, we transfected truncated variants of ANXA2 into 293T cells followed by co-IP analysis, which indicated that NEDD4L could directly bind to the Annexin 1 domain of ANXA2 (Fig. 5N, O). Collectively, both *LINC00941* and NEDD4L interacted with the Annexin 1 domain, leading to a competitive interaction with ANXA2, which indirectly reduced the ubiquitination of ANXA2 by NEDD4L.

### ANXA2 activates FAK/AKT signaling to promote proliferation, migration, and invasion

**Fig. 6:**
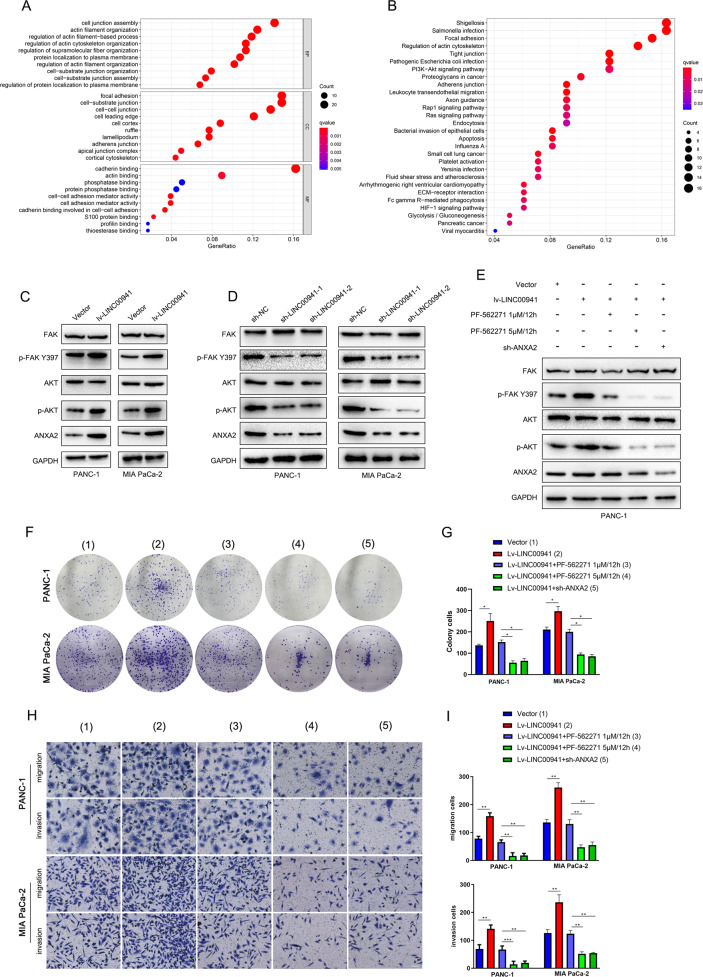
ANXA2 activates FAK/AKT signaling promoting proliferation, migration, and invasion. **A**, **B** Gene Ontology (GO) and Kyoto Encyclopedia of Genes and Genomes (KEGG) analysis based on TCGA data to predict the potential function and involved signaling pathway of ANXA2 by using R software. **C** The protein expressions of FAK, p-FAK, AKT, p-AKT, and ANXA2 in the empty vector group and LINC00941 overexpression group were measured by western blot analysis. **D** The protein expressions of FAK, p-FAK, AKT, p-AKT, and ANXA2 in the negative control group and LINC00941 knockdown group were measured by western blot analysis. **E** Western blot was used for analyzing the protein expressions of FAK, p-FAK, AKT, p-AKT, and ANXA2 in empty vecotr group, lv-LINC00941 group, lv-LINC00941 combining PF-562271 (an inhibitor of FAK pathway) group and lv-LINC00941 + sh-ANXA2 group, respectively. **F**–**I** Colony-formation assay and transwell assay were performed to proliferation, migration, and invasion abilities in the empty vector group, lv-LINC00941 group, lv-LINC00941 + PF-562271 group and lv-LINC00941 + sh-ANXA2 group. *, **, and ***, respectively, represent *P* < 0.05, *P* < 0.01, and *P* < 0.001.

We demonstrated that *LINC00941* functions as a decoy to competitively interact with ANXA2 and upregulate its expression. High expression of ANXA2 could promote PC progression. To investigate the mechanism by which ANXA2 affected the malignant phenotype, we used Gene Ontology (GO) and Kyoto Encyclopedia of Genes and Genomes (KEGG) analyses according to TCGA data to predict their potential function and the involved signaling pathway. The results indicated that the main function of ANXA2 was the regulation of the actin cytoskeleton, focal adhesion, and PI3K–AKT signaling (Fig. 6A, B). To clarify this prediction, we evaluated critical markers in the FAK/AKT signaling pathway including FAK, phosphorylated FAK (p-FAK) at tyrosine 397, AKT, and phosphorylated AKT (p-AKT) at serine 473. The results confirmed that *LINC00941* dramatically promoted ANXA2 expression as well as inducing increased p-FAK and p-AKT, implying that *LINC00941* might promote PC progression via activating the FAK/AKT signaling axis (Fig. 6C, D). To further clarify that LINC00941 regulates ANXA2-mediated activation of FAK/AKT signaling, we used different concentrations of PF-562271, which is a potent, reversible, and ATP-competitive inhibitor of FAK, to inhibit the activation of FAK, as examined by western blot analysis. Notably, PF-562271 substantially suppressed the expression of p-FAK and p-AKT and blocked the activation of FAK/AKT signaling, which was concentration-dependent. In addition to PF-562271, silencing *ANXA2* had the same inhibitory effect (Fig. 6E). Subsequently, we measured proliferation, migration, and invasion when we added PF-562271 or knocked down *ANXA2* in PANC-1 and MIA PaCa-2 cells infected with lv-LINC00941. The results illustrated that PF-562271 treatment and *ANXA2* silencing both inhibited proliferation, migration, and invasion. Taken together, the data demonstrated that *LINC00941* upregulated ANXA2 and activated FAK/AKT signaling.

### *LINC00941* promotes PC cell proliferation and metastasis through upregulating ANXA2 and activating FAK/AKT signaling in vivo

**Fig. 7:**
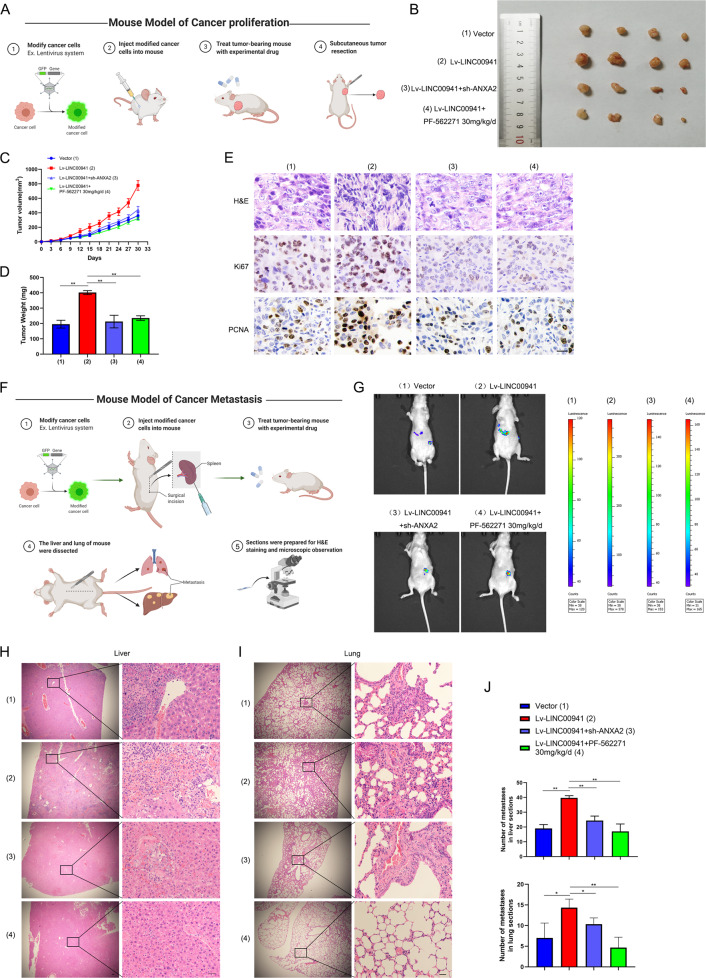
LINC00941 promotes PC growth and metastasis through upregulating ANXA2 and activating FAK/AKT signaling in vivo. **A** The schematic diagram of the mouse model of cancer proliferation. **B** The photograph of tumors in empty vector group, lv-LINC00941 group, lv-LINC00941 + sh-ANXA2 group and lv-LINC00941 + PF-562271 (30 mg/kg/d) group (*n* = 4/per group). **C** The tumor volume in these groups. **D** The tumor weight in these groups. **E** IHC analysis was performed to evaluate the expression of proliferation markers, including Ki67 and PCNA, and grade malignancy using H&E staining. **F** The schematic diagram of the mouse model of cancer metastasis. **G** The living imaging of tumors in empty vector group, lv-LINC00941 group, lv-LINC00941 + sh-ANXA2 group and lv-LINC00941 + PF-562271 (30 mg/kg/d) group (*n* = 4/per group). **H** The liver metastases in these groups were stained with H&E. **I** The lung metastases in these groups were stained with H&E. **J** The statistical chart of liver or lung metastases. * and **, respectively, represent *P* < 0.05 and *P* < 0.01.

**Fig. 8:**
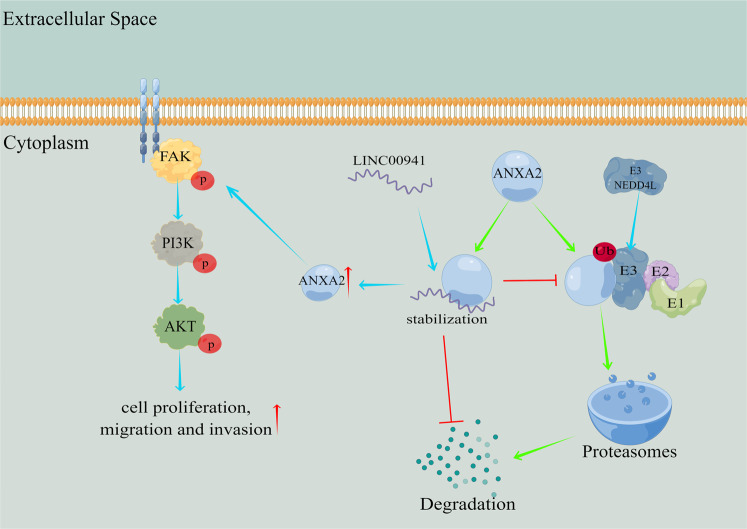
The schematic diagram of LINC00941 promoting pancreatic cancer progression. LINC00941 acts as a decoy to interact with ANXA2, blocking the key interaction domain between NEDD4L and ANXA2, inhibiting ubiquitin-mediated degradation of ANXA2. Eventually, LINC00941 stabilizes ANXA2 and promotes activation of FAK/AKT signaling, resulting in facilitating pancreatic cancer proliferation, migration, and invasion (the figure was drawn by Figdraw, ID:UPATW888a4).

To better determine the malignant function of *LINC00941*, we applied subcutaneous tumorigenesis and spleen metastasis models to evaluate growth and metastasis in vivo. As shown in Fig. 7A, we injected PANC-1 cells infected with lv-LINC00941 or lv-LINC00941 plus *ANXA2* knockdown into the subcutaneous area of the armpit. After tumor-bearing, mice underwent treatment with PF-562271 orally. The results suggested that *LINC00941* significantly promoted tumor growth (Fig. 7B), with the largest tumors recorded in the *LINC00941* group (Fig. 7C, D). However, PF-562271 treatment and *ANXA2* silencing both partially suppressed the promotion of tumor growth by *LINC00941* overexpression (Fig. 7B–D). Meanwhile, Ki67 and PCNA, two critical markers of proliferation, were also most highly expressed in the lv-LINC00941 group compared with the control and treatment groups. PF-562271 and *ANXA2* silencing could partially reversed the results (Fig. 7E). In the metastasis model shown in Fig. 7F, luciferase lentivirus combined with lv-LINC00941 or lv-LINC00941 plus *ANXA2* knockdown was co-infected into PANC-1 cells, and the cells were injected into the spleen, then treated with PF-562271. In vitro imaging indicated *LINC00941* dramatically promoted liver metastasis (Fig. 7G). Microscopy of liver and lung sections suggested that *LINC00941* also promoted metastasis formation in liver and lung. However, to a certain extent, PF-562271 and *ANXA2* silencing suppressed the *LINC00941*-mediated metastasis (Fig. 7H–J). Collectively, the above evidence suggested that *LINC00941* promoted PC tumor growth and metastasis in vivo through upregulating ANXA2 and activating FAK/AKT signaling (Fig. 8).

## Discussion

LncRNAs are emerging as important factors in the malignant progression of tumors, including PC [30]. In our previous study, we found that *LINC00941* was overexpressed in PC and could act a sponge of *miR-335-5p* to elevate ROCK1 expression, thus activating the LIMK1/Cofilin-1 signaling pathway. Notably, cytoplasmic lncRNAs were reported to bind some crucial proteins to exert their biological functions [31]. Recently, *LINC00941* was reported to bind STE20-like protein kinase 1 (MST1) [12], SMAD4 [32], CAPRIN2 [33], and SPRR5 [34] in different tumors, increasing target protein expression, stability, and activity. In this study, we found that *LINC00941* could promote the stabilization of ANXA2 and suppress its ubiquitination. Meanwhile, *ANXA2* silencing or NEDD4L overexpression could partly reversed the function of *LINC00941* in PC proliferation and migration. These results demonstrated that LINC00941 might regulate PC progress via binding key protein and modulating its stablization.

Protein ubiquitination is a critical post-translation modification in various disease processes, including cancer. In colorectal cancer, *SNHG17* lncRNA inhibited PES1 ubiquitination and degradation via blocking the interaction of Trim23 and PES1 [35]. Jiang et al. reported that *RP11-286H15.1* lncRNA bound to PABPC4 via 620–750 nucleotides of RP11-286H15.1 and ubiquitinated PABPC4 via TRIM37 in hepatocellular carcinoma [36]. Therefore, lncRNAs located in the cytoplasm probably interact with ubiquitinase enzymes to regulate the stability and expression of the target protein, thus exerting a tumor-promoting or tumor-suppressing role. Then, we explored the critical sequence and domain in the interaction of *LINC00941*, ANXA2, and NEDD4L. On the basis of the immunoprecipitation experiment, we found that *LINC00941* probably interacted with ANXA2, moreover, nucleotides 500–1390 of *LINC00941* and the Annexin 1 domain were required for their binding. Meanwhile, in *LINC00941*-overexpressing PC cells, ANXA2 ubiquitylation was significantly inhibited, and protein stability was upregulated. These results indicated that *LINC00941* probably acted as a decoy by inhibiting ANXA2 ubiquitylation and promoting protein stability. A previous study reported that TRIM65 promoted bladder urothelial carcinoma progress via ubiquitination of ANXA2 [37]. Furthermore, in esophageal cancer, ANXA2 expression was overexpressed and promoted the tumor progression by activating the MYC/HIF1A/VEGF signaling pathway [38]. ANXA2 acted an as an oncogene in hepatocellular carcinoma progression, which was modified by SIRT6/UBE3A [39]. Therefore, ANXA2 probably exerts an oncogenic role in various tumor types, and ubiquitination of ANXA2 might be a critical regulatory process in tumorigenesis. Following this train of thought, we further identified the essential ubiquitinase enzyme involved in *LINC00941*-mediated ANXA2 deubiquitination, NEDD4L was screened among predicted candidates. Interestingly, NEDD4L and *LINC00941* both interacted with the Annexin 1 domain of ANXA2, thus regulating ANXA2 stability and expression. Lee et al. reported that NEDD4L is a tumor suppressor gene and interacts with ULK1 to inhibit autophagy and growth in PC [40]. Meanwhile, NEDD4L was upregulated by NDRG1, which is a tumor suppressor gene in PC, inhibiting the PI3K and RAS pathway [41]. Finally, a rescue experiment indicated that NEDD4L and ANXA2 probably acted as downstream targets in the process of *LINC00941* promotion of PC proliferation and metastasis. These results illustrated that *LINC00941* promoted PC progression via inhibiting the interaction of NEDD4L with ANXA2 and the ubiquitination of ANXA2.

To clarify the role and mechanism of ANXA2 in PC progression, bioinformatic analysis indicated that ANXA2 probably activates the AKT and FAK signaling pathways. Meanwhile, previous studies suggested that ANXA2 may be related to the AKT and FAK signaling pathways. ANXA2 was reported to activate AKT/GSK3β and AKT/mTOR signaling in gastric, breast, and colorectal cancer [42–44], whereas ANXA2 is a substrate of ITSN1-L-mediated cell–substrate adhesion through the FAK/integrin β3 pathway [45]. Therefore, we validated the effect of NEDD4L in the activation of AKT and FAK signaling. Current research findings indicated that the FAK and AKT signaling pathways are the critical signaling cascades in the onset of PC, and thus their biological functions are extremely broad, including metabolic reprogramming, immune escape, abnormal division, and enhanced invasion and metastasis. The high activation of FAK/AKT signaling in PC results in its critical role in the malignant progression of tumor cells. Therefore, there have been some important advances based on FAK or AKT as a targeted therapy in PC. However, as the underlying mechanism by which AKT regulate PC progression has not been fully studied, the treatment method targeting AKT has not yet been clinically applied [46, 47]. The current literature suggests that FAK-targeted therapy can play a vital anti-cancer effect in some tumor subtypes and can also enhance the efficacy of other treatment methods [48]. Furthermore, we confirmed that FAK inhibitor PF-562271 could effectively reverse the role of *LINC00941* in promoting PC cell progression. On the basis of the above experimental results, we can conclude that the *LINC00941*/NEDD4L/ANXA2 signaling axis ultimately mediates the progression of PC through the activation of the FAK/AKT signaling pathway in PC.

## Conclusion

We discovered that in pancreatic cancer, *LINC00941* acts as a protein decoy in the cytoplasm and confirmed that it can competitively bind to the E3 ubiquitin ligase NEDD4L to inhibit the ubiquitination of ANXA2 and promote its stabilization. At the same time, *LINC00941* promoted the upregulation of ANXA2 expression, activated the FAK and AKT signaling pathways, and ultimately promoted the malignant progression of PC.

## Supplementary information

Ssupplementary Figure and Table Legends

supplementary Table 1

supplemental Figure 1

supplemental Figure 2

supplemental Figure 3

aj-checklist

Original Data File

## Data Availability

All data generated and analyzed during this study are included in this published article and are available on request.
