# Supplementary material for: LINC00941 promotes pancreatic cancer malignancy by interacting with ANXA2 and suppressing NEDD4L-mediated degradation of ANXA2
Source: Cell Death Dis. 2022 Aug 18;13(8):718. doi: 10.1038/s41419-022-05172-2 (PMC9385862; doi:10.1038/s41419-022-05172-2)
Supplement: Supplementary file 1 — Ssupplementary Figure and Table Legends [file 41419_2022_5172_MOESM1_ESM.docx]

**Supplementary Figure and Table legends.**

**Supplementary Table 1. The sequences involved in this study**.

**Supplementary Figure 1. LINC00941 was remarkably associated with poor prognosis.** (a) Kaplan–Meier curve analyzed the overall survival in stage I. (b) Kaplan–Meier curve analyzed the overall survival in stage II. (c) Kaplan–Meier curve analyzed the overall survival in grade I. (d) Kaplan–Meier curve analyzed the overall survival in grade II. (e) Kaplan–Meier curve analyzed the overall survival in grade III. The independent risk factor of prognosis involved in LINC00941 expression,age, gender, grade and stage were analyzed using univariate (f) and multivariate (g) regression analysis. (h) The correlation between LINC00941 and immune cell infiltration from Timer 2.0 (http://timer.cistrome.org/), which is based on TCGA database.

**Supplementary Figure 2. LINC00941 interacted with ANXA2.** (a) Bioinformation method (RNA Interactome Database, http://www.rnainter.org/) identified the potential target genes. (b) The results of mass spectrometry from RNA pull-down by the sense and antisense of LINC00941. The protein (c) and mRNA (d) expression of ANXA2 in five pancreatic cancer cell lines compared to ANXA2.

**Supplementary Figure 3. ANXA2 interacted with NEDD4L.** (a) The results of mass spectrometry from proteins immunoprecipitated by IgG and anti-ANXA2. (b) Bioinformation method (UbiBrowser, http://ubibrowser.ncpsb.org.cn/ubibrowser/) identified the potential target E3 ligase.
