# Supplementary material for: LINC00941 promotes pancreatic cancer malignancy by interacting with ANXA2 and suppressing NEDD4L-mediated degradation of ANXA2
Source: Cell Death Dis. 2022 Aug 18;13(8):718. doi: 10.1038/s41419-022-05172-2 (PMC9385862; doi:10.1038/s41419-022-05172-2)
Supplement: Supplementary file 2 — supplementary Table 1 [file 41419_2022_5172_MOESM2_ESM.docx]

**Supplemental Table 1. The sequences involved in this study**.

| Gene | Sequence (5’—3’) | |
| --- | --- | --- |
| *LINC00941* | Forward | CTTCTCTGAACTGCGGCTCA |
|  | Reverse | GGCCTCCTTGCTGACTGATT |
| ANXA2 | Forward | GAGCGGGATGCTTTGAACATT |
|  | Reverse | TAGGCGAAGGCAATATCCTGT |
| GAPDH | Forward | CTGGGCTACACTGAGCACC |
|  | Reverse | AAGTGGTCGTTGAGGGCAATG |
