# Supplementary figures and images for: LINC00941 promotes pancreatic cancer malignancy by interacting with ANXA2 and suppressing NEDD4L-mediated degradation of ANXA2
Source: Cell Death Dis. 2022 Aug 18;13(8):718. doi: 10.1038/s41419-022-05172-2 (PMC9385862; doi:10.1038/s41419-022-05172-2)

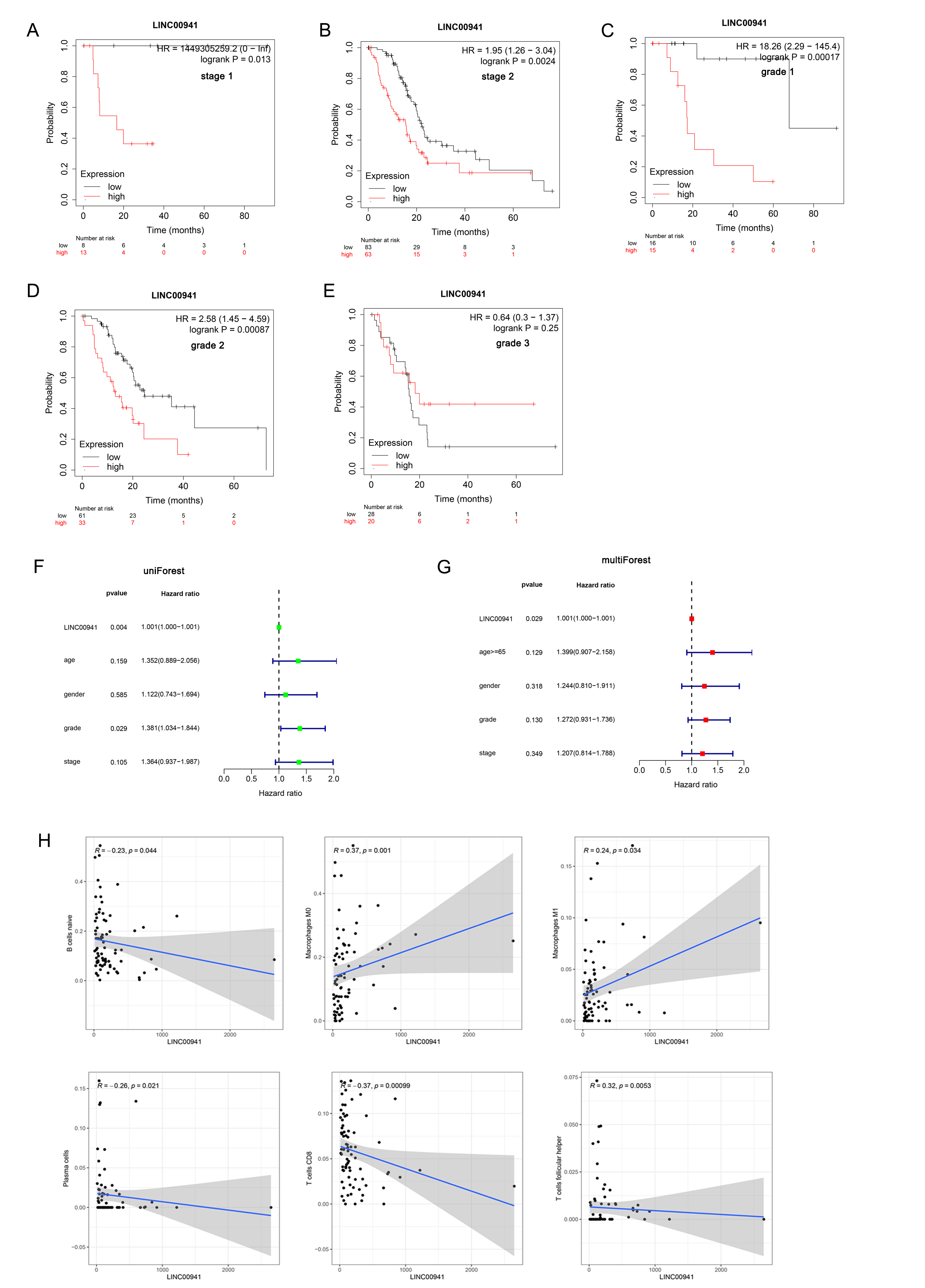

Supplement: Supplementary file 3 — supplemental Figure 1 [file 41419_2022_5172_MOESM3_ESM.tif]

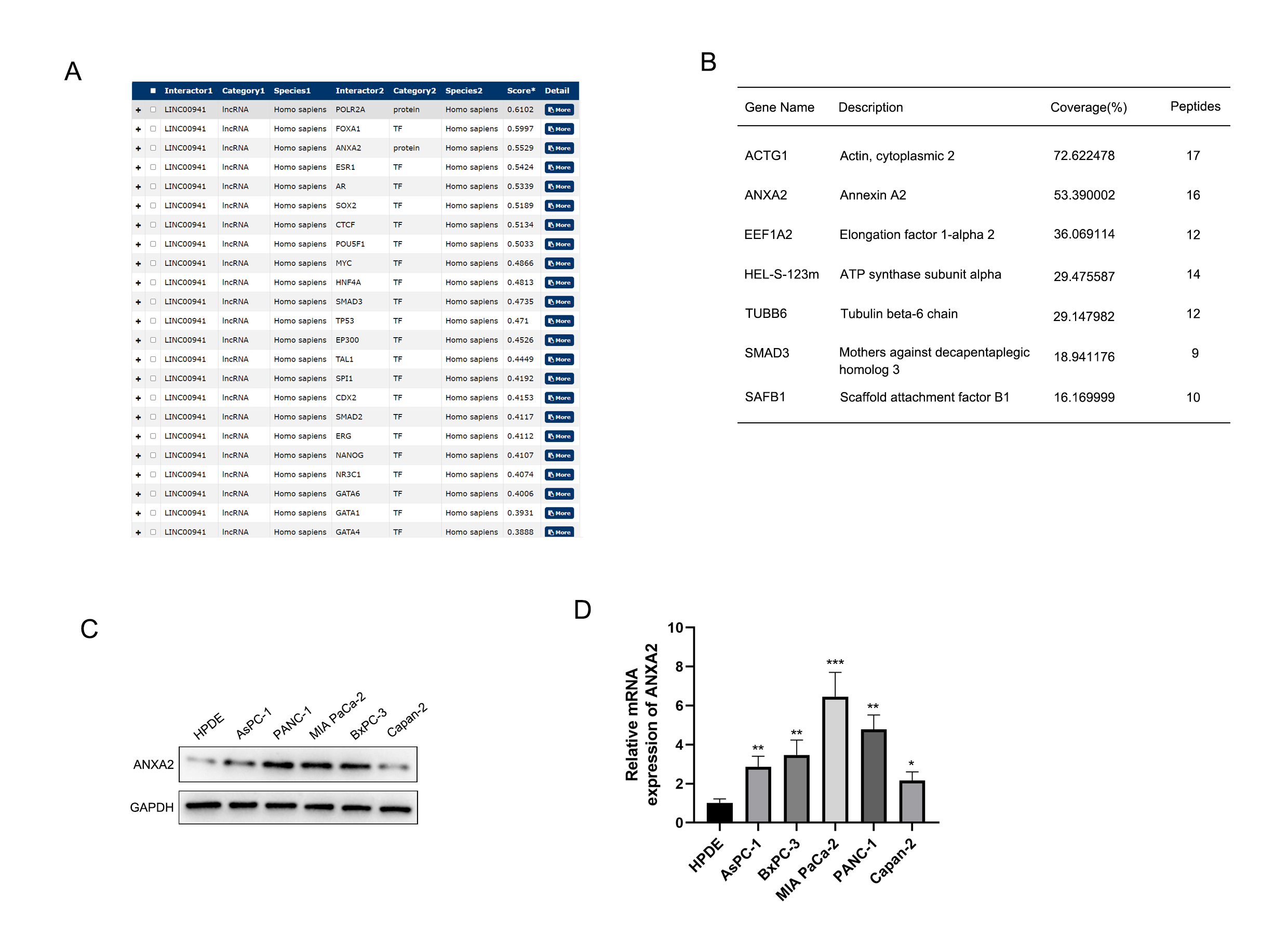

Supplement: Supplementary file 4 — supplemental Figure 2 [file 41419_2022_5172_MOESM4_ESM.tif]

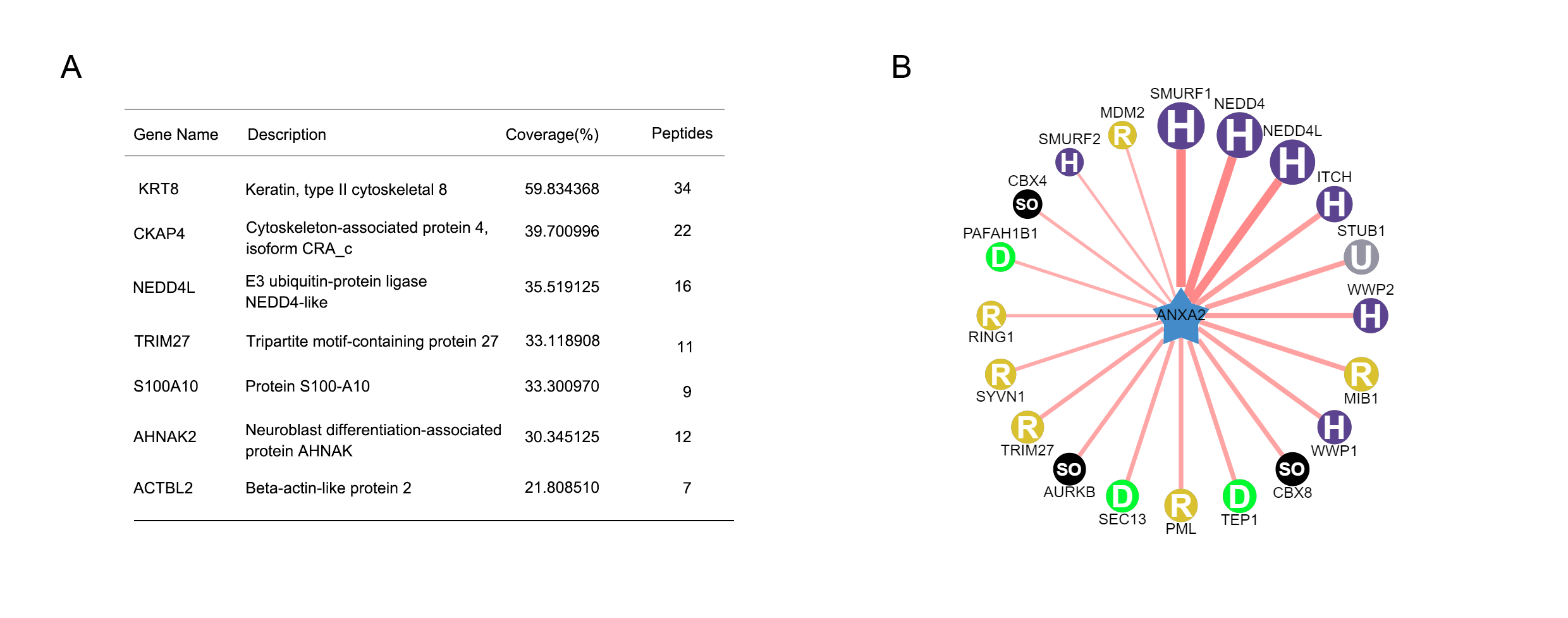

Supplement: Supplementary file 5 — supplemental Figure 3 [file 41419_2022_5172_MOESM5_ESM.tif]
