# Supplementary material for: LINC00941 promotes pancreatic cancer malignancy by interacting with ANXA2 and suppressing NEDD4L-mediated degradation of ANXA2
Source: Cell Death Dis. 2022 Aug 18;13(8):718. doi: 10.1038/s41419-022-05172-2 (PMC9385862; doi:10.1038/s41419-022-05172-2)

Figure 2C

ANXA2

36kDa

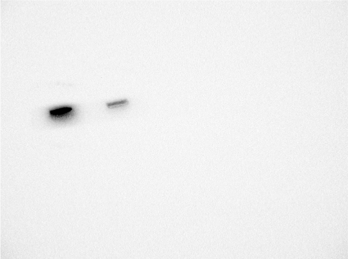

ANXA2

36kDa

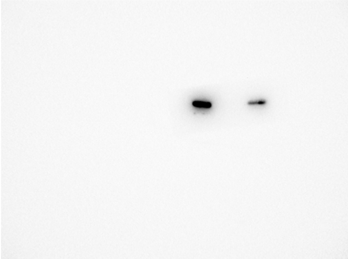

Figure 2G

ANXA2

36kDa

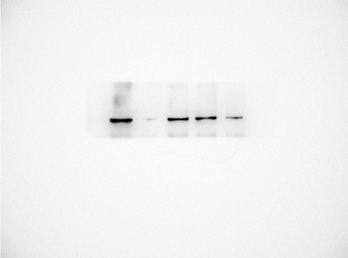

Figure 2I

ANXA2

36kDa

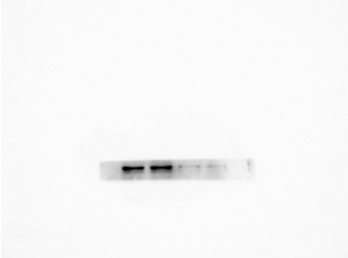

supplemental Figure 2C

ANXA2

36kDa

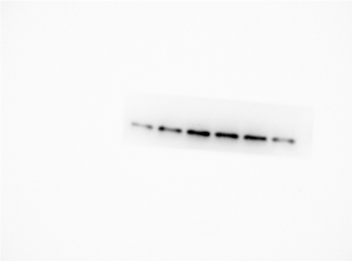

GAPDH

36kDa

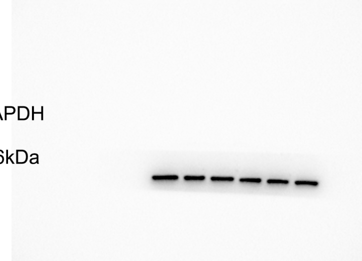

Figure 2D

ANXA2
36kDa

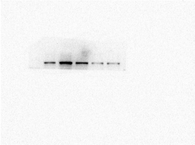

β-actin
42kDa

ANXA2
36kDa

β-actin
42kDa

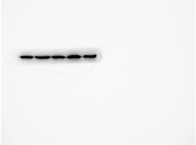

Figure 2F

ANXA2
36kDa

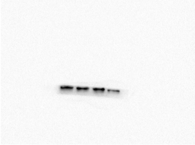

GAPDH
36kDa

ANXA2
36kDa

GAPDH
36kDa

Figure 2H

ANXA2
36kDa

GAPDH
36kDa

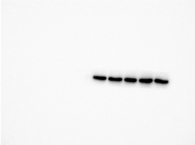

ANXA2
36kDa

GAPDH
36kDa

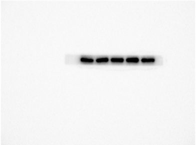

Figure 2J

ANXA2
36kDa

GAPDH
36kDa

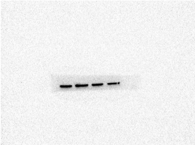

ANXA2
36kDa

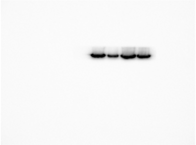

GAPDH
36kDa

Figure 2L

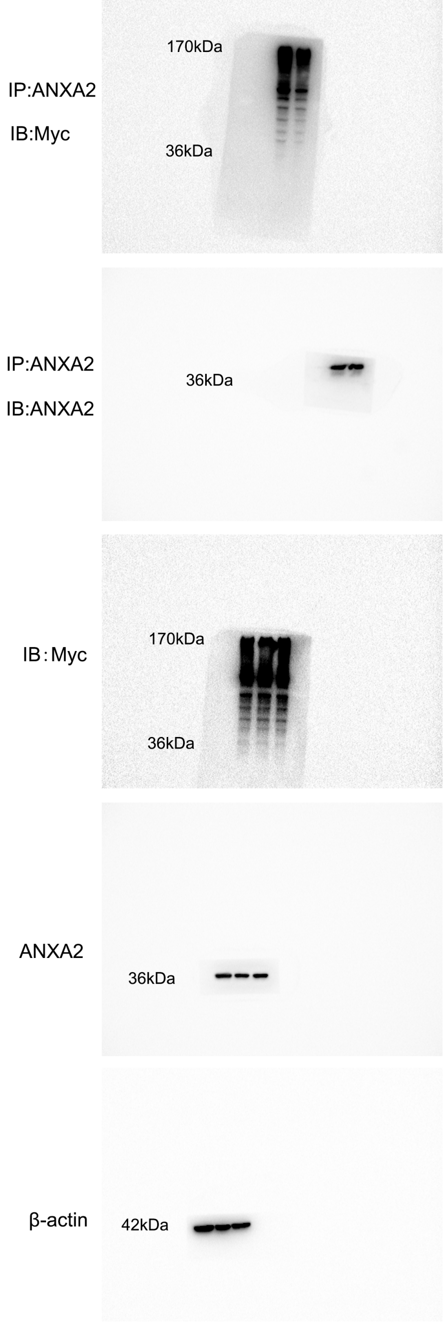

Figure 2M

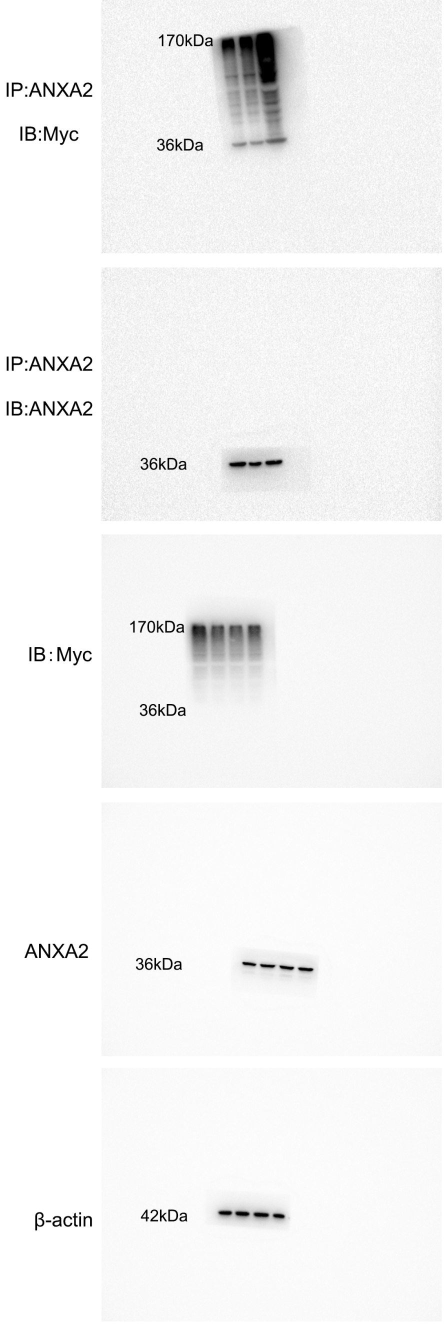

Figure 3A

ANXA2
36kDa

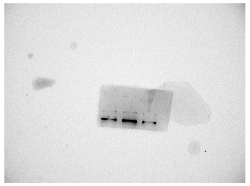

ANXA2
36kDa

GAPDH
36kDa

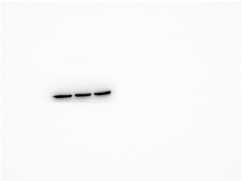

GAPDH
36kDa

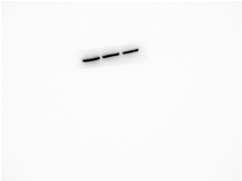

Figure 3F

ANXA2
36kDa

ANXA2
36kDa

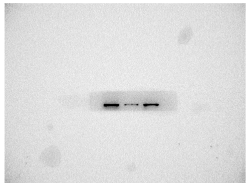

GAPDH
36kDa

GAPDH
36kDa

Figure 5B

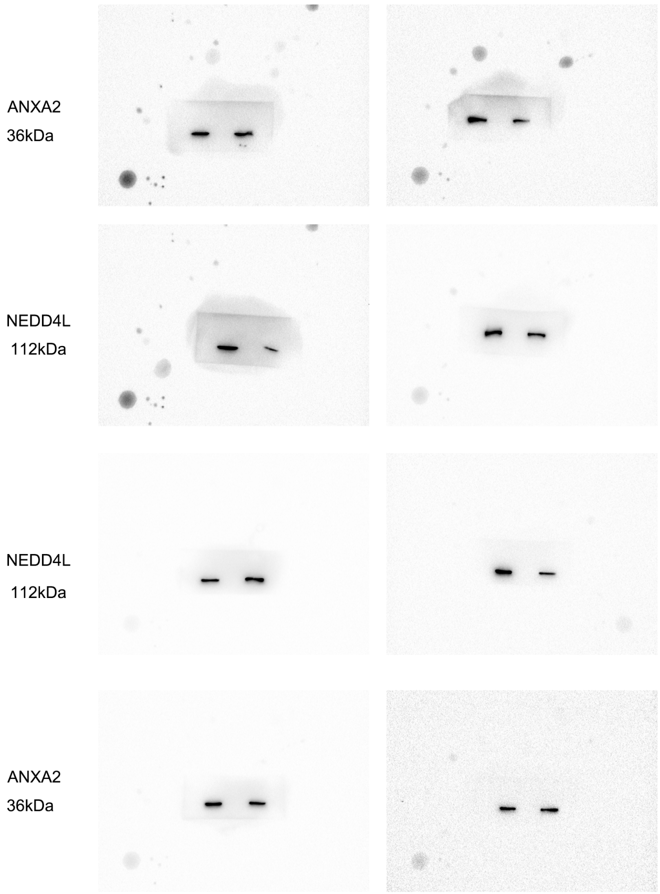

Figure 5C

HA
125kDa

Flag
45kDa

HA
125kDa

Flag
45kDa

GAPHD
36kDa

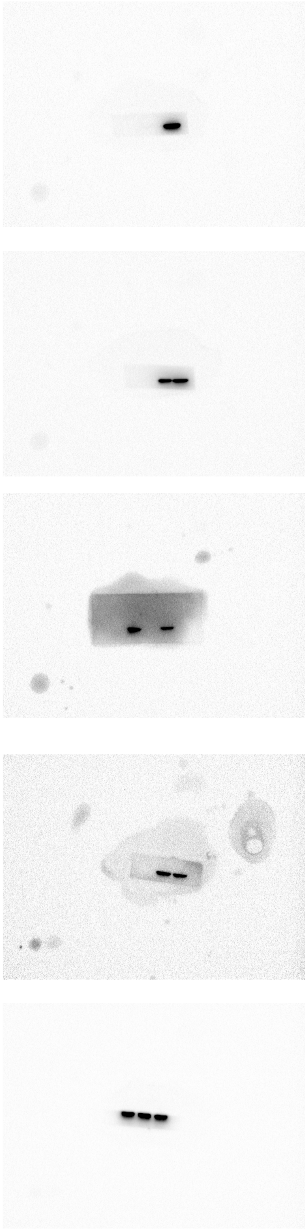

Figure 5D

HA
125kDa

Flag
45kDa

HA
125kDa

Flag
45kDa

GAPHD
36kDa

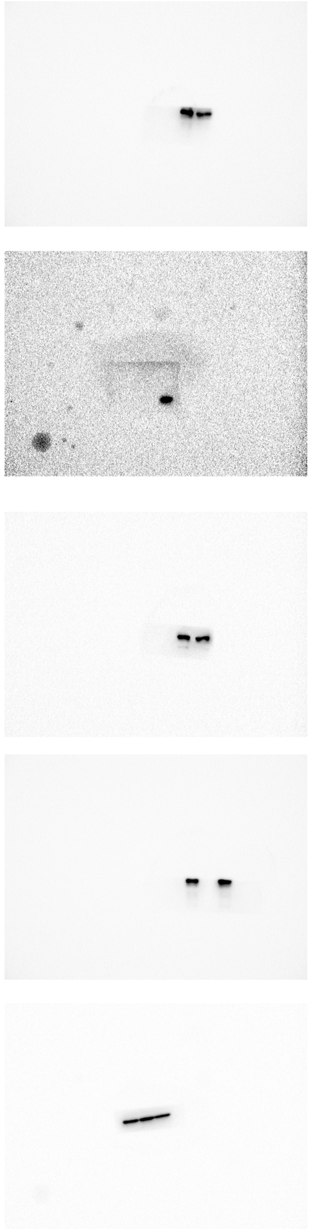

Figure 5E

NEDD4L
112kDa

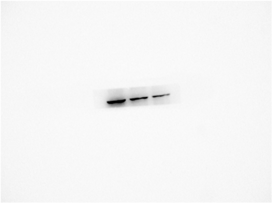

NEDD4L
112kDa

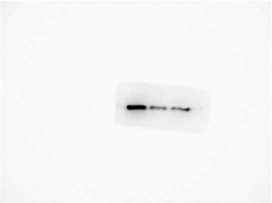

ANXA2
36kDa

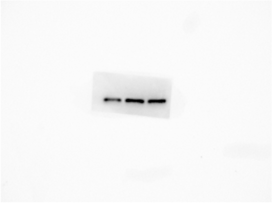

ANXA2
36kDa

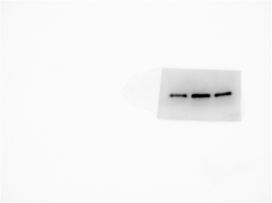

GAPDH
36kDa

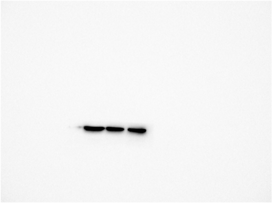

GAPDH
36kDa

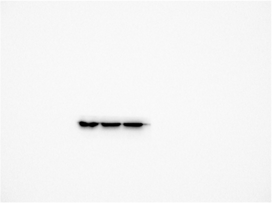

Figure 5F

170kDa
Myc
36kDa

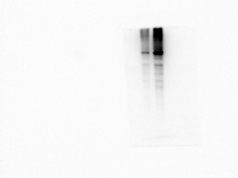

170kDa
Myc
36kDa

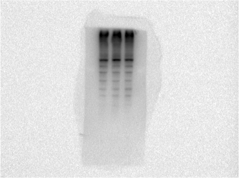

Flag
45kDa

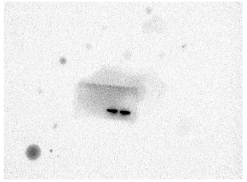

Flag
45kDa

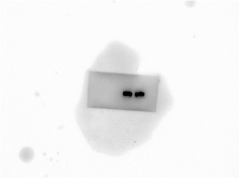

GAPDH
36kDa

HA
125kDa

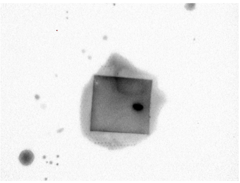

Figure 5G

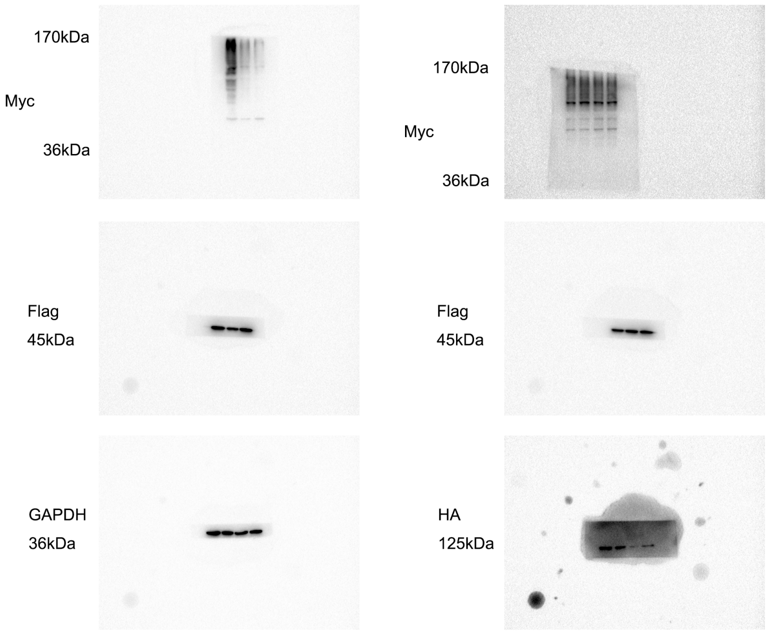

Figure 5H

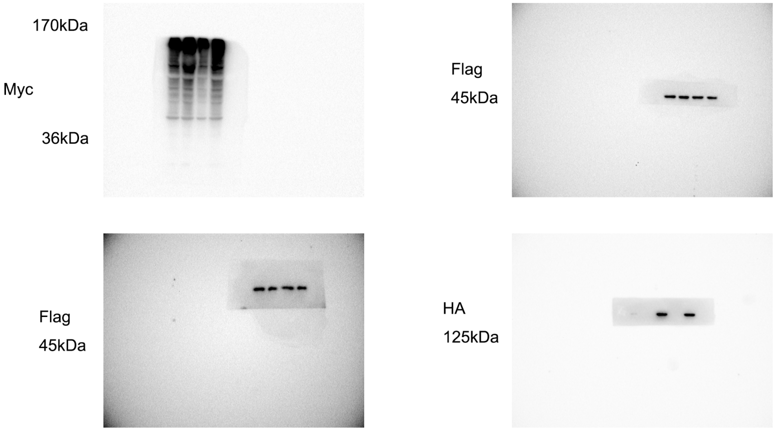

Figure 5I

170kDa
Myc
36kDa

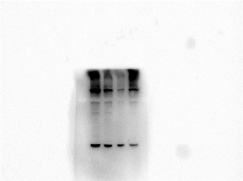

Flag
45kDa

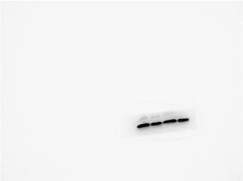

Flag
45kDa

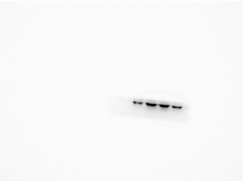

NEDD4L
112kDa

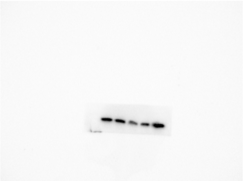

Figure 5K

Flag
45kDa

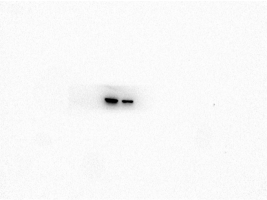

HA
125kDa

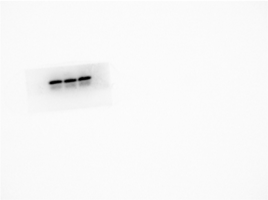

HA
125kDa

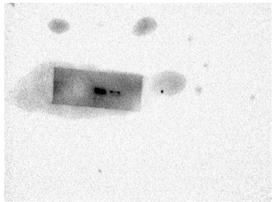

Flag
45kDa

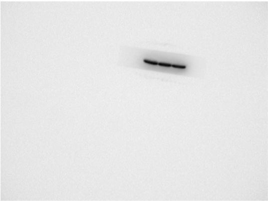

HA
125kDa

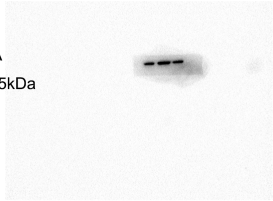

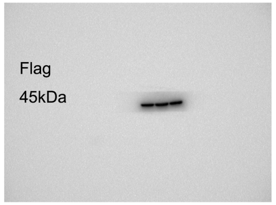

Flag
45kDa

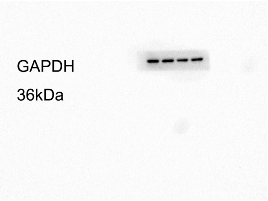

GAPDH
36kDa

Figure 5J

Flag
45kDa

HA
125kDa

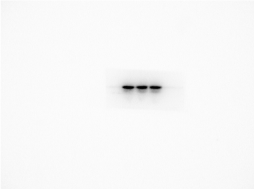

HA
125kDa

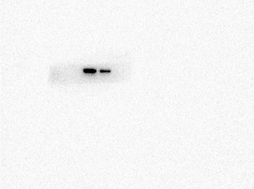

Flag
45kDa

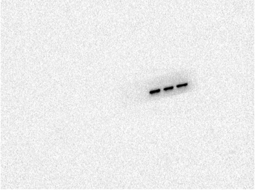

HA
125kDa

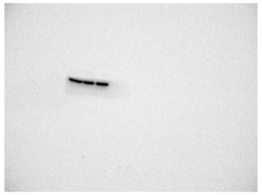

Flag
45kDa

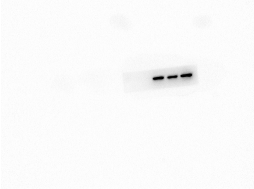

GAPDH
36kDa

Figure 5L

ANXA2
36kDa

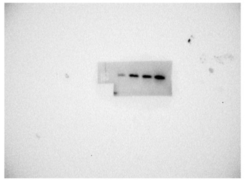

ANXA2
36kDa

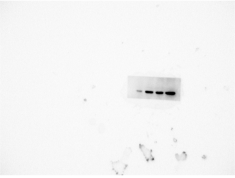

NEDD4L
112kDa

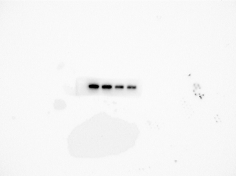

NEDD4L
112kDa

GAPDH
36kDa

GAPDH
36kDa

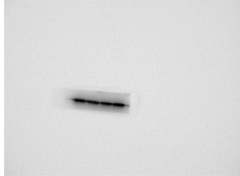

Figure 5M

ANXA2
36kDa

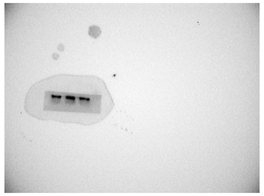

ANXA2
36kDa

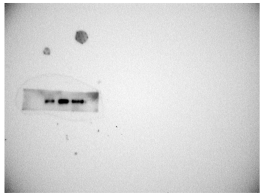

HA
125kDa

HA
125kDa

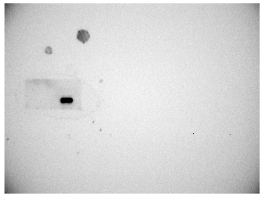

GAPDH
36kDa

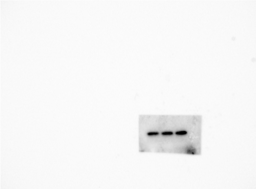

GAPDH
36kDa

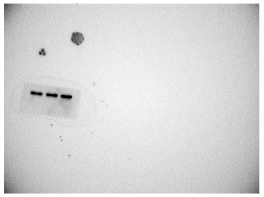

Figure 5N

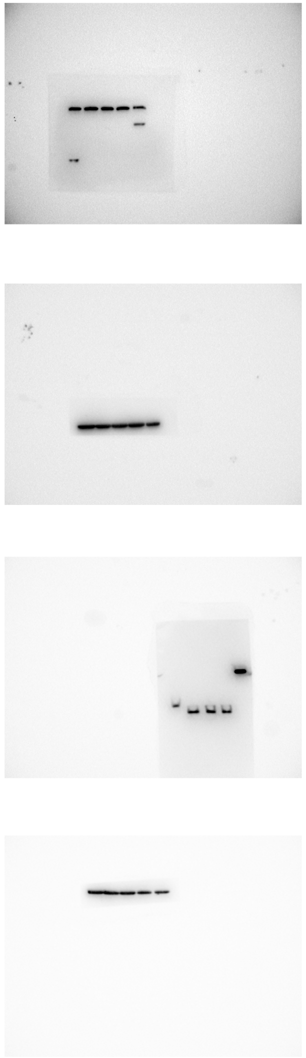

Flag 55kDa 45kDa 20kDa

HA 125kDa

Flag 45kDa 20kDa 15kDa

HA 125kDa

Figure 5O

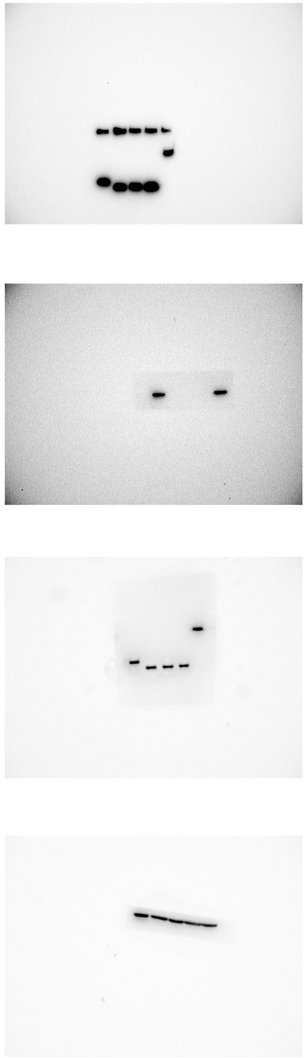

Flag 55kDa 45kDa 20kDa 15kDa

HA 125kDa

Flag 45kDa 20kDa 15kDa

HA 125kDa

Figure 6C

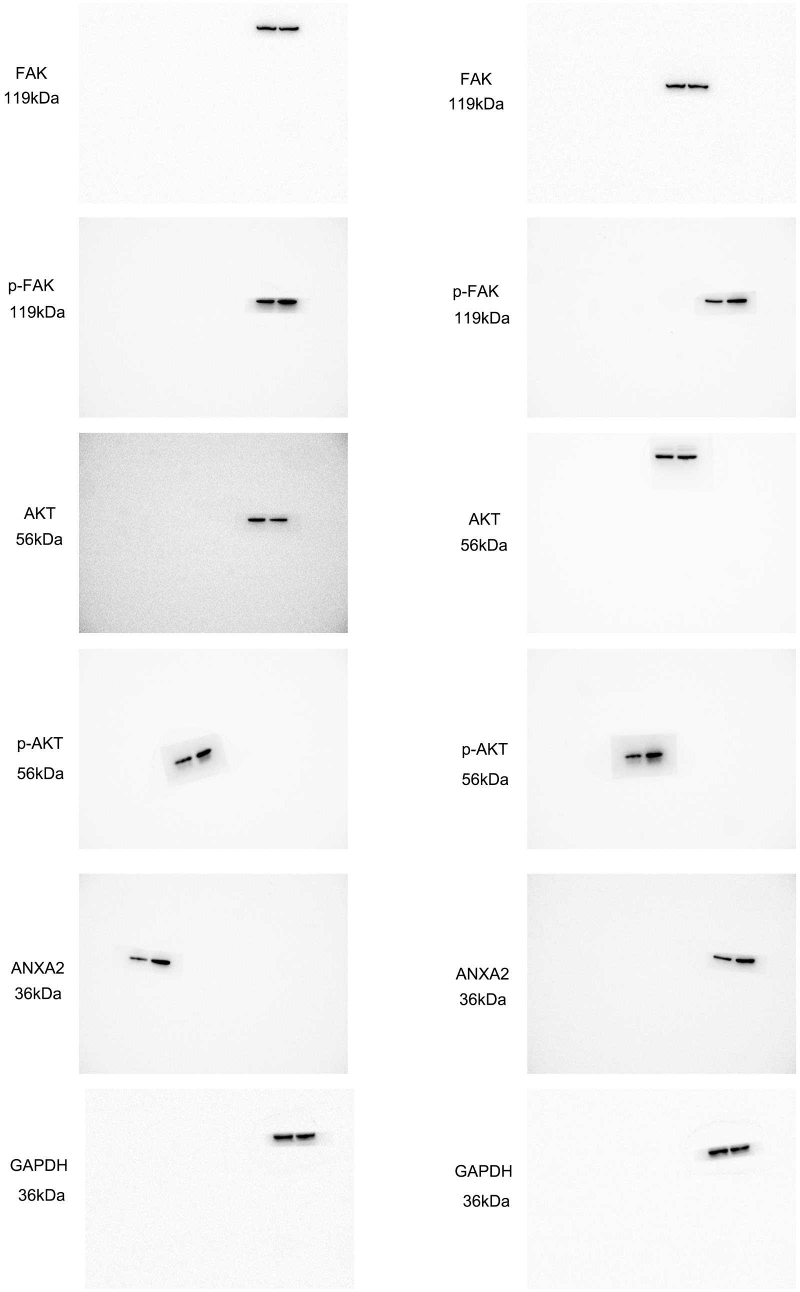

FAK
119kDa
FAK
119kDa
p-FAK
119kDa
p-FAK
119kDa
AKT
56kDa
AKT
56kDa
p-AKT
56kDa
p-AKT
56kDa
ANXA2
36kDa
ANXA2
36kDa
GAPDH
36kDa
GAPDH
36kDa

Figure 6D

FAK
119kDa

p-FAK
119kDa

AKT
56kDa

p-AKT
56kDa

ANXA2
36kDa

GAPDH
36kDa

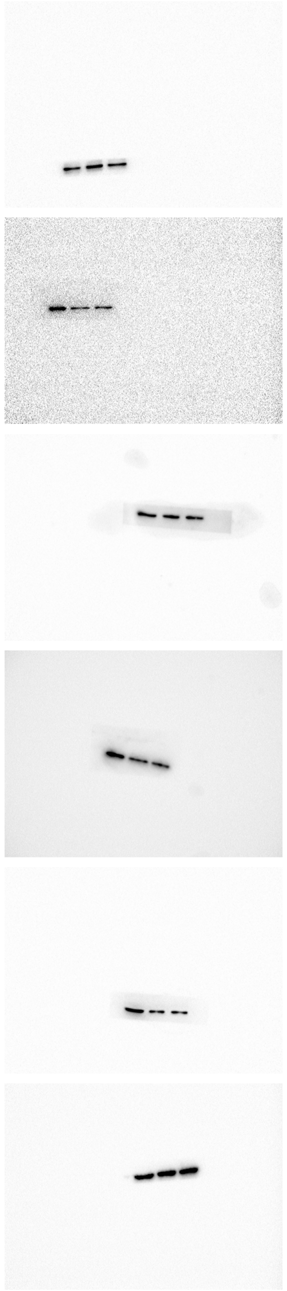

FAK
119kDa

p-FAK
119kDa

AKT
56kDa

p-AKT
56kDa

ANXA2
36kDa

GAPDH
36kDa

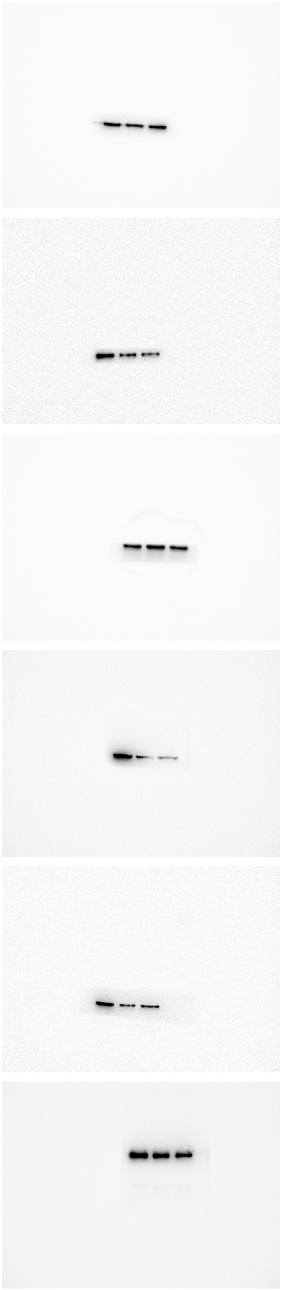

Figure 6E

FAK
119kDa

p-FAK
119kDa

AKT
56kDa

p-AKT
56kDa

ANXA2
36kDa

GAPDH
36kDa

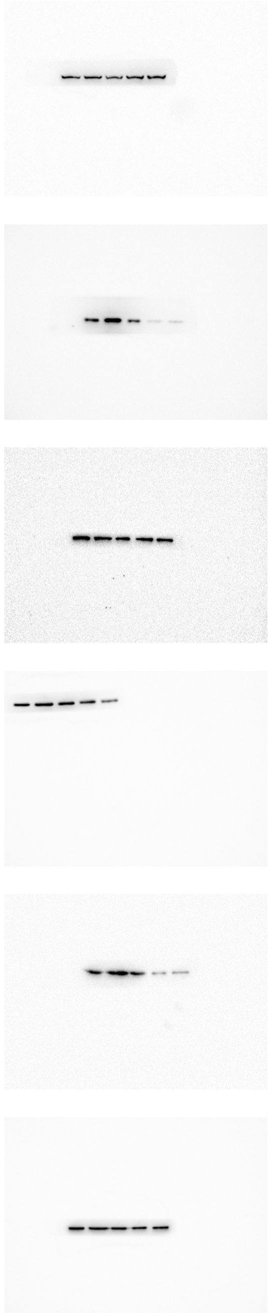

Supplement: Supplementary file 7 — Original Data File [file 41419_2022_5172_MOESM7_ESM.pdf]
